# Biomimetic Synapses Based on Halide Perovskites for Neuromorphic Vision Computing: Materials, Devices, and Applications

**DOI:** 10.1007/s40820-025-02052-0

**Published:** 2026-02-09

**Authors:** Zhongwen Sun, Xuan Zhao, Haonan Si, Qingliang Liao, Yue Zhang

**Affiliations:** 1 Academy for Advanced Interdisciplinary Science and Technology, Key Laboratory of Advanced Materials and Devices for Post-Moore Chips Ministry of Education, Beijing Key Laboratory for Advanced Energy Materials and Technologies, University of Science and Technology Beijing, Beijing, 100083 People’s Republic of China; 2 School of Materials Science and Engineering, State Key Laboratory for Advanced Metals and Materials, University of Science and Technology Beijing, Beijing, 100083 People’s Republic of China

**Keywords:** Perovskites, Biomimetic synapses, Neuromorphic devices, Neuromorphic vision computing

## Abstract

The demand for accurate perception of the physical world has led to a dramatic increase in visual sensing data, accompanied by challenges in the energy efficiency of data processing. However, conventional vision systems with separated sensor and processing units struggle to handle increasingly intricate and large-scale data. As such, a rethinking of architecture design is necessary. Inspired by human visual systems, neuromorphic vision computing systems in which computation tasks are moved partly to the sensory or memory units offer transformative solutions to these challenges. As crucial hardware support, biomimetic synapses that replicate synaptic functions and dynamics are urgent for the development of future computing, while further progress requires materials that can support synaptic weight modulation. Given their excellent optical, electrical, and ion migration properties, halide perovskite materials have emerged as promising candidates for biomimetic synapses. Here we review the latest efforts of synaptic devices based on halide perovskite materials for neuromorphic vision computing. We demonstrate the operating mechanism of perovskite synapses and introduce their potential applications in realizing neuromorphic vision computing. We address challenges and future directions related to biomimetic perovskite synapses.

## Highlights

- Insightful discussion of the unique properties of perovskite materials in terms of optical, electrical, and ion migration properties, along with extensively analysis of different categories of perovskite materials for biomimetic synapses.
- Comprehensive exploration of the structures and working mechanisms of perovskite synapses, emphasizing their transformative opportunities in neuromorphic vision computing.
- Prospective outlook on the approach to the performance optimization methods of synaptic devices, covering material optimization, device structure design, and external physical signal regulation.

## Graphical abstract

**Figure:**
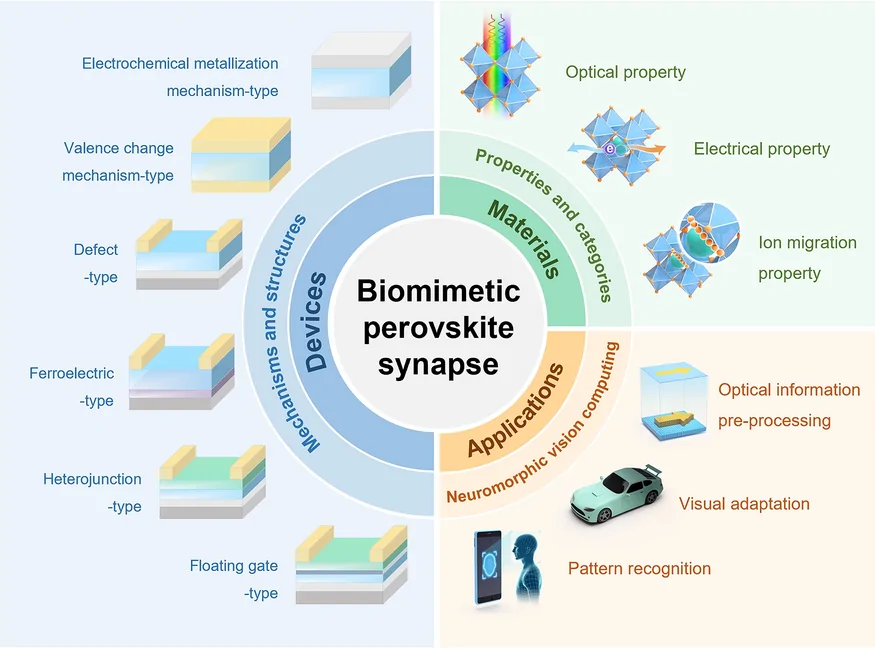

## Introduction

Vision is known as the primary way for artificial systems to perceive and engage with the environment, as it can collect a wide range of information with excellent adaptability [1]. Artificial vision systems have provided impressive performance in fields such as autonomous systems, security, and manufacturing, significantly contributing to the development of advancement of artificial intelligence [2]. However, against the backdrop of increasingly complex and extreme real-world scenarios, coupled with the explosive growth of image data, the upgrading of conventional artificial vision systems, which rely on the von Neumann architecture, has hit a bottleneck [3, 4]. This is attributed to the frequent transmission of excessive redundant data caused by the separated architecture composed of sensors, memory, and processing components [5, 6]. It is thus necessary to rethink the design of artificial vision systems to improve their performance and efficiency [7].

The tightly interconnected and energy-efficient human visual system offers a promising solution. Human visual system is responsible for perceiving and processing over 80% of the information handled by the human body, while also exhibiting low redundancy, low power consumption, and strong robustness [8]. This benefits largely from the hierarchical structure formed by the retina and the visual cortex of the human brain. Generally speaking, the retina is responsible for perceiving external light signals, converting them into electrical signals, and transmitting them to the brain [9]. The human brain, with its 10^11^ neurons interconnected through 10^15^ synapses, functions as a dynamic and reconfigurable processor to simultaneously store and compute vast amounts of intricate, unstructured information with extremely low energy consumption [10]. Biological synapse, the critical junctions linking neurons, act as the fundamental physical substrates that endow the brain with adaptive and learning capabilities [11]. They exhibit various forms of synaptic plasticity, such as short-term plasticity (STP) and long-term plasticity (LTP), enabling the brain to efficiently process information and perform parallel operations [12].

Inspired by such hierarchical structure, neuromorphic vision computing systems has gained prominence [13]. By reducing unnecessary data transfer between sensors and post-processors, neuromorphic vision computing can notably boost both data processing speed and energy efficiency [14]. Fundamentally, biomimetic synaptic devices that implement synaptic functions at the device level represent an essential step for realizing such architecture [15]. A variety of materials have been proposed for simulating synaptic functions in synaptic devices, each presenting distinct advantages. For instance, two-dimensional (2D) materials are promising candidates for making synaptic devices because they allow for the tuning of structural and electronic properties via phase engineering [16, 17]. Metal oxide materials are currently the most popular choice for synaptic devices, as they offer designable metastable states, CMOS compatibility, and exceptional stability [18–20]. Organic semiconductors hold the unique advantages of flexibility, biological compatibility, and cost-effectiveness solution processability [21, 22]. Halide perovskite, merging the merits of inorganic and organic semiconductors, has recently triggered great interest [23, 24]. They offer a number of valuable optoelectronic properties needed for achieving synaptic functionality such as high absorption coefficients, tunable bandgaps, high carrier mobility, and long carrier diffusion lengths [25, 26]. They also exhibit unique ion migration capability, enabling their wide application in memristive devices with the potential for mimicking synapses [27]. Besides, they are compatible with cost-effective solution processes, offering practical advantages for scaling [28, 29]. To date, perovskites have been utilized in a variety of synaptic devices, yielding promising initial results.

Here, we present a comprehensive assessment of biomimetic synapses based on halide perovskites, focusing on aspects from materials, devices to applications. We share our viewpoint regarding the unique properties of halide perovskites for biomimetic synapses and elaborate on fundamental materials selection for realizing highly functional biomimetic synapses. Furthermore, we categorize typical perovskite synapses into several groups according to their device structures and explain their working mechanism. Emerging applications in neuromorphic vision computing of perovskite synapses are then highlighted. Finally, we extensively discuss remaining challenges and point out strategies to further promote developments in the field.

## Fundamentals of Perovskite Materials

Halide perovskites have emerged as promising candidates for neuromorphic applications, possessing a synergistic combination of excellent optical, electrical, and ion migration properties that enables the fabrication of high-performance synaptic devices [30]. In terms of optical properties, perovskites exhibit a large light absorption coefficient, implying that a film just a few hundred nanometers thick can absorb the vast majority of incident light [31, 32]. This ensures high-light capture efficiency and enabling the fabrication of devices with very thin layers [26, 33]. They also exhibit a wide spectral absorption range that can be easily tuned by altering the composition and dimension of the perovskite materials [34, 35]. These characteristics support optical modulation of synaptic weights, making them suitable for optoelectronic or even all-optical synapses. Regarding electrical properties, their charge carrier transport characteristics are favorable [36]. Their low-to-medium carrier mobility in the dark ensures low initial current, while significantly higher mobility under light excitation boosts postsynaptic current, enabling devices with wide dynamic range and multiple conductance states [37]. Additionally, their long carrier lifetime contributes to increased carrier diffusion length and extended synaptic retention time [38]. Notably, halide perovskites exhibit a high degree of ion mobility, providing a basis for designing synaptic devices. Their high defect tolerance and low point defect formation energy allow stable crystal structures even with high defect densities [30]. Meanwhile, ions in halide perovskites are prone to migrate due to their low migration activation energy (e.g., 0.2–0.4 eV for MAPbI_3_ compared to 1.5 eV or even higher for metal oxides like HfOₓ) [39, 40]. This enable memristive behavior via ion migration-induced conductive filaments or interface energy barrier changes [41, 42]. Ion concentration and migration ability can be regulated by composition (e.g., metal halide bond energy, lattice structure) and external stimuli (light, voltage), with light further reducing migration activation energy and modulating defect density via photogenerated carrier recombination [30, 43]. These properties facilitate fast-switching, low-voltage synaptic devices capable of encoding temporal information, establishing perovskites as exceptional candidates for biomimetic synapses.

**Fig. 1:**
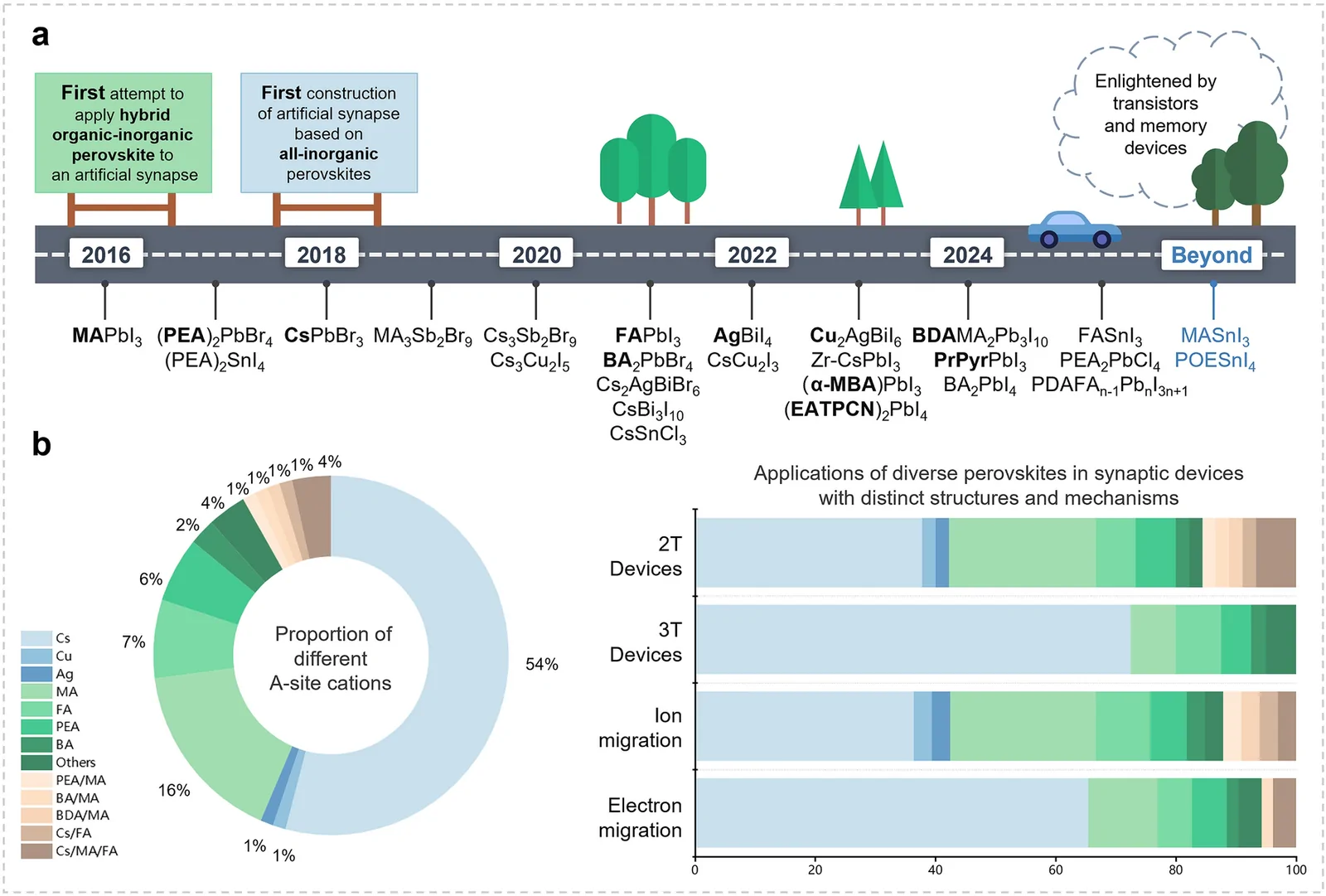
Perovskite materials for biomimetic synapses.** a** Developmental history of perovskite materials for synaptic devices. **b** Applications of diverse perovskites in synaptic devices with distinct structures and mechanisms

Fundamentally, metal halide perovskites are a type of semiconductor material with the general formula ABX_3_, where A represents a monovalent cation, which can be organic cations such as MA^+^ and FA^+^, or inorganic cations such as cesium (Cs^+^). B-site is typically occupied by divalent metal ions such as lead (Pb^2+^) and tin (Sn^2+^), and X represents halide anions such as iodide (I^−^), chloride (Cl^−^), or bromide (Br^−^) [44, 45]. Depending on the type of A-site ions, perovskites can be categorized into two main groups: hybrid organic–inorganic perovskites and all-inorganic perovskites, each offering unique advantages for synaptic functionality [46]. A historical timeline of the progress of biomimetic synapse based on these two types of perovskites from 2016 up to the present is presented in Fig. 1a, b. It can be observed that there has been an upward trend of the utilization of organic–inorganic hybrid perovskite materials. Meanwhile, the design of organic molecules has emerged as a highly effective and popular approach. Representative examples are listed below to track the current development status of synaptic devices based on organic–inorganic hybrid and all-inorganic perovskites from a compositional perspective.

Organic–inorganic hybrid perovskites possess exceptional electronic properties, rendering them suitable materials for two-terminal synaptic devices based on ion migration mechanisms [47, 48]. In 2016, Xiao et al. confirmed the potential of MAPbI_3_ to achieve synaptic functions. Taking advantage of the ionic conductor properties and the convenience of solution processability, a synaptic device with the structure of Au/MAPbI_3_/indium tin oxide (ITO)/poly (3,4-ethylenedioxythiophene)-poly(styrenesulfonate) (PEDOT:PSS) was easily established by spin coating. The ion migration led to the formation of a switchable p-i-n structure, thereby enabling the device to exhibit memory behavior [49]. Since then, a variety of organic–inorganic hybrid perovskites have been extensively investigated in the field of synaptic devices research. Gong et al. developed a FAPbI_3_-based synaptic device that not only demonstrated various essential synaptic behaviors, such as excitatory postsynaptic current (EPSC) and paired pulse facilitation (PPF), but also showed that the value of EPSC can be regulated by the controlling the intensity of light irradiation [50]. In addition to the commonly used MA- and FA-based perovskite materials, the pioneering work of Kagan et al. in the use of (PEA)_2_ SnI_4_ as a channel layer for perovskite transistors may unveil novel opportunities [51]. Zhu et al. demonstrated high-performance transistors with a MASnI_3_-based channel layer, obtaining a high hole mobility of approximately 20 cm^2^ V^−1^ s^−1^ as well as an on/off current ratio greater than 10^7^, with a threshold voltage (V_TH_) of 0 V [52]. A pure tin perovskite thin-film transistor (TFT) based on a CsFAPEA triple-cation combination was subsequently reported, exhibiting a hole mobility exceeding 70 cm^2^ V^−1^ s^−1^ [53]. This value is comparable to that of commercial TFT devices based on low-temperature polycrystalline silicon. Given the superior high mobility exhibited by these perovskite devices, it is also feasible to create perovskite synaptic devices that store optical information by integrating photons as supplementary regulatory terminals.

All-inorganic perovskites display superior stability and are typically employed as the photosensitive layer in three-terminal synaptic devices based on electron migration mechanisms [54–56]. Among these, 0D perovskite quantum dots (QDs) exhibit enhanced optical response and tunable bandgap, encouraging their utilization in synaptic devices [57, 58]. CsPbBr_3_ QDs are among the most commonly used in this field. In one of the earliest study in 2018, Wang et al. employed CsPbBr_3_ QDs as a light-absorbing floating-gate layer to achieve photo-programming operations and also succeeded in simulating synaptic behaviors such as spike-rate-dependent plasticity (SRDP), STP, and LTP [59]. Additionally, CsPbBr_3_ is also compatible with other simple solution-based techniques (such as inkjet printing and screen printing) for the fabrication of large-area and high-quality films [60]. This makes it possible to construct highly integrated flexible and ultrathin synaptic devices rapidly and cost-effectively in mass production. This potential was initially validated in the study of Shi et al., which they employed a printing method to fabricate a 2, 7-dioctyl [1]-benzothieno[3,2-b][1]benzothiophene (C8-BTBT) /poly(styrene) (PS)/CsPbBr_3_ QDs ternary synaptic device [61]. In addition to Pb-based perovskites, concerns regarding toxicity have prompted research efforts toward the preparation of perovskites using alternative elements. The excellent visible-light absorption of single-crystal CsBi_3_I_10_ perovskite was utilized by Huang et al. to enhance the photo-response of the device and the synaptic plasticity of the response. The favorable stability of the Bi-based perovskite was reflected in the unencapsulated devices’ capacity to maintain stability in air for a 30 day period [62]. The aforementioned properties also highlight the appeal of all-inorganic perovskites as promising candidates for manufacturing electronic devices capable of withstanding extremely harsh environmental conditions. Efforts have also been dedicated to the development of Ag-based and Cu-based all-inorganic halide perovskite synaptic devices, which demonstrate a diverse range of synaptic behaviors [63, 64].

In general, metal halide perovskites merge the appealing qualities of excellent optical, electrical, and ion migration properties, in conjunction with solution processability, enabling diverse devices to perform synaptic functions. Their versatility in composition has led to a rich and colorful library of perovskite materials, supporting varied synaptic functions [26, 34]. Perovskites have demonstrated remarkable capabilities in biomimetic synapses, holding potential for keeping in step with the latest information technology revolution.

## Categories of Perovskite Synapses

**Fig. 2:**
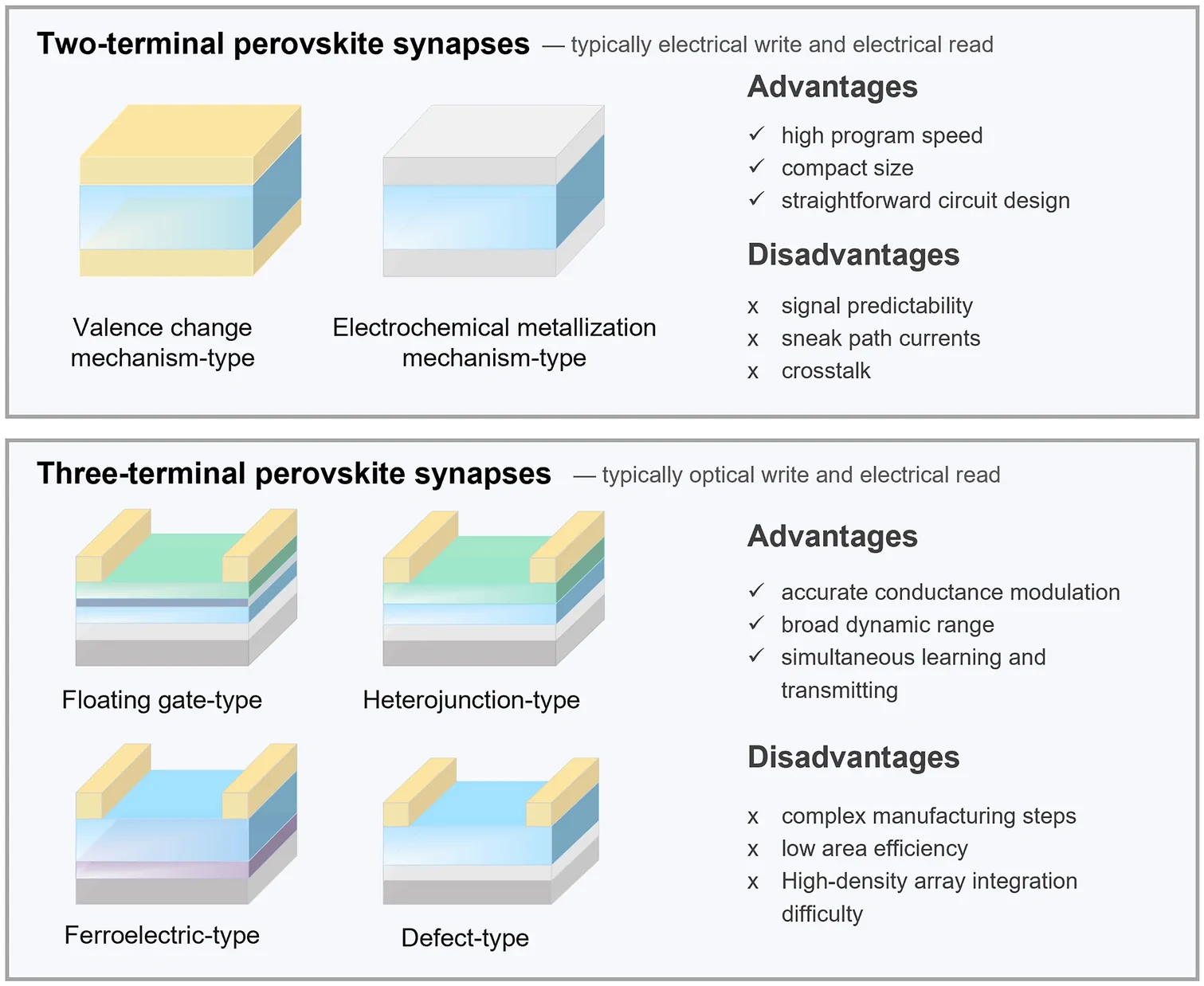
Categories of perovskite synapses and the corresponding advantages and disadvantages

Benefiting from the inherent solution processability and component tunability, halide perovskites offer a myriad of opportunities to design biomimetic synaptic devices with varied device architectures. Depending on the terminal number, perovskite synaptic devices can be classified into two-terminal (2 T) devices and three-terminal (3 T) devices, as shown in Fig. 2. Diverse device architectures enable a variety of operating mechanisms, thereby providing the requisite synaptic plasticity for neuromorphic applications. Typically, 2 T synaptic devices refer to electrical synapses programmable through electrical stimulation. They are characterized by the integration of memory and computing, which inherently aligns with the information processing characteristics of the biological brain. And 3 T synaptic devices are categorized as optoelectronic synapses optical signals as the additional modulation method. Benefiting from non-contact optical signals, optoelectronic synapses boast advantages such as fast response speed, low energy consumption, and low crosstalk [65, 66]. More importantly, against the inevitable trend of the continuous integration of artificial intelligence and sensor technology, optoelectronic synapses with the capability to conduct local data processing and decision-making represent an important simulation of the human visual system [67]. In this chapter, recent advancements of synaptic devices based on these two structures are summarized, and conductance change mechanism of each type is elaborated in detail.

### Two-Terminal Perovskite Synapses

2 T perovskite synapses feature a simple configuration, analogous to biological synapses, which consists of a top electrode (presynaptic terminal), a bottom electrode (postsynaptic terminal), and an active layer in its middle region (synaptic cleft). Devices with this straightforward design offer the benefit of easy fabrication and easy miniaturization [15]. For 2 T perovskite synaptic devices, the resistance transition arises from the formation of conductive filaments and alteration of interface barriers due to ion migration induced by applied electric field. Based on their operating mechanisms, these devices can be further categorized into valence change mechanism (VCM) type and electrochemical metallization mechanism (ECM) type [68].

#### Valence Change Mechanism Type

With regard to the VCM type, mobile ions are typically halide ions and their vacancies that move easily within the lattice, thanks to the soft inorganic lattice and low ion migration activation energy. There are usually two approaches to achieving conductance modulation via ion migration, the filamentary type, and the interfacial type [69].

For the filamentary type, the typical resistance switching process is demonstrated in a perovskite synaptic device based on lead-free inorganic perovskite Cu_2_AgBiI_6_ [64]. The voltage scanning curve indicated that the device exhibited a transition from high-resistance state (HRS) to low-resistance state (LRS) regardless of whether a positive or negative sweep voltage was applied. This bipolar resistance transition corresponds to the formation and rupture process of conductive filaments formed by iodine vacancies. Iodine vacancies were initially dispersed throughout the perovskite space. Upon reaching a specific threshold of external voltage, these vacancies aggregated to form filaments, resulting in a decrease in resistance. Subsequently, under the influence of reverse voltage stimulation, these formed filaments underwent a fracture process, thus leading to resistance recovery. Furthermore, in addition to the filaments composed of X-site ions mentioned above, B-site ions and other defects, such as inverse positions, can also actively participate in the modulation of resistance states. A VCM mechanism triggered by the migration of bromine vacancies has also been reported [70]. The authors discovered an initial low-resistance state even without applied voltage. They proposed that thermal annealing-induced generation of V_Br_ could trigger a redox reaction leading to reduction of interstitial Sb^3+^ to metallic Sb, potentially contributing to the formation of initial conducting filaments. In a perovskite synapse with the configuration of Ag/ polymethyl methacrylate (PMMA)/(PrPyr)PbI_3_/PEDOT:PSS/ITO, the authors emphasized that, except for the relatively bulky PrPyr^+^ cation and the ions I^−^ and Pb^2+^, defects such as vacancies, interstitials, and anti-sites also played a significant role in the resistive switching process [71].

To circumvent the inherent randomness of filament-type synaptic devices caused by filament rupture, interfacial type synaptic devices have been fabricated. A preliminary study on this mechanism was conducted by John et al. [72]. They proposed a hypothesis whereby negatively charged bromide anions and A-site cation vacancies would drift and accumulate in proximity to the interface between the hole-transporting material, resulting in self-p-doping. Conversely, the positively charged A-cations and bromide vacancies would result in n-doping of the interface between the electron-transporting material. The doping phenomenon ultimately facilitated the injection of carriers. Upon the removal of the pulsed voltage, the presence of a built-in electric field as well as the ion concentration gradient, caused ionic reverse diffusion/relaxation. This phenomenon modulated the carrier injection barrier, consequently affecting the device conductance.

**Fig. 3:**
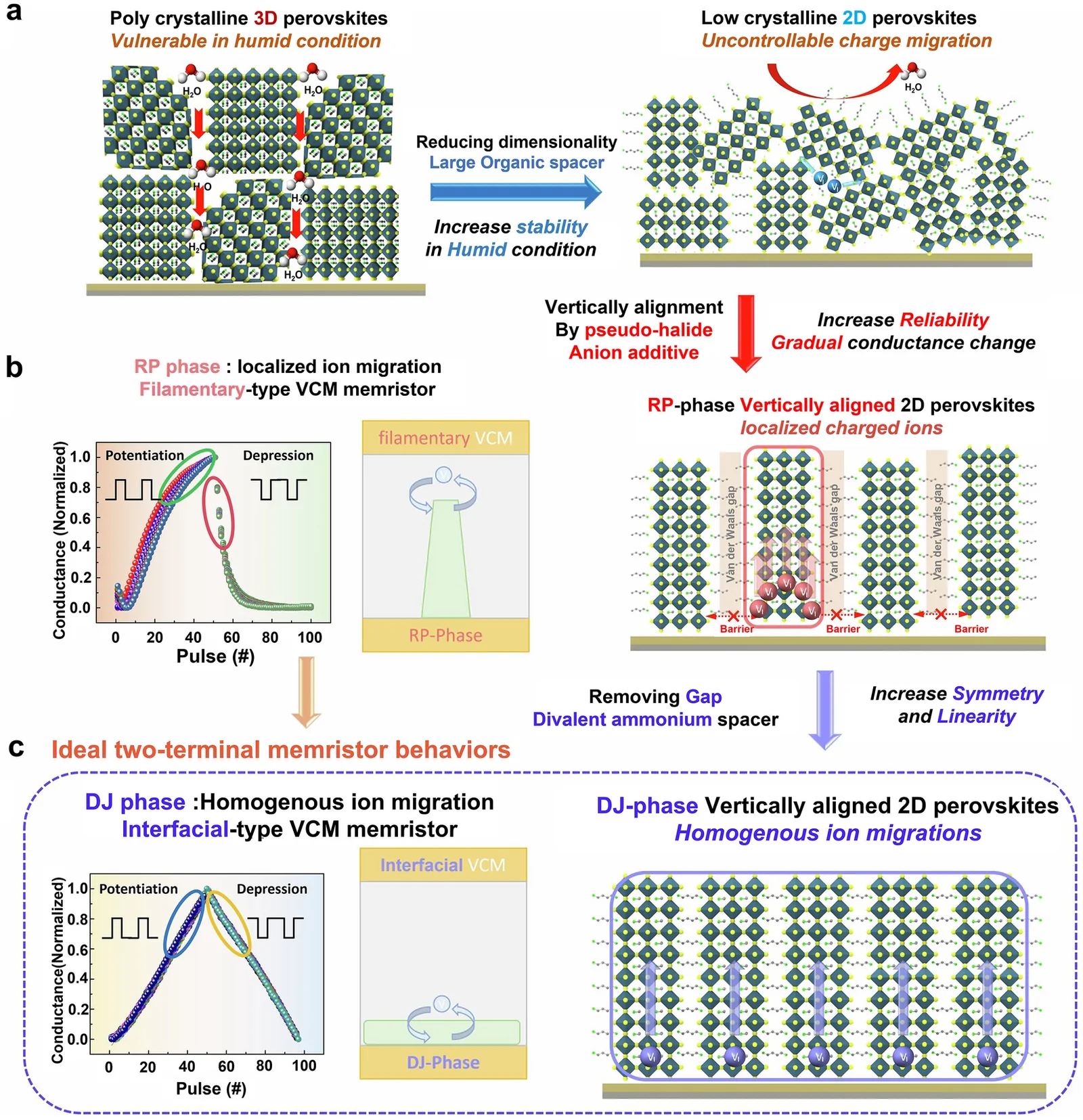
**a** Improvement of moisture stability of 3D perovskite by addition of hydrophobic large organic cations. **b** Schematic illustration of filamentary mechanism and synaptic behavior of RP-phase perovskite-based memristors. **c** Schematic illustration of interfacial mechanisms and almost perfect synaptic behavior of DJ-phase perovskite-based memristors. Reproduced from Ref. [73] with permission from Springer Nature, Copyright 2024

Subsequently reported interfacial type perovskite synaptic devices have demonstrated impressive stable resistance switching behavior [73]. Given the influence of the polycrystalline nature of three-dimensional (3D) perovskites on their water stability, it was considered that two-dimensional perovskites featuring large organic cations are considered to exhibit more dependable conductance programming. By incorporating pseudo-halide additives, a structure where 2D perovskites are vertically aligned perpendicular to the substrate can be achieved. This vertically aligned perovskite improved poor ion mobility and uncontrolled ion migration, thereby attaining reliable synaptic behavior (Fig. 3a**)**. Vertically oriented synaptic devices based on Ruddlesden–Popper (RP) phase perovskite BA_2_MA_n-1_PbnI_3n+1_ and Dion–Jacobson (DJ) phase perovskite BDAMA_n-1_Pb_n_I_3n+1_ were fabricated. As shown in Fig. 3b, c, almost perfect linear and symmetrical LTP and long-term depression (LTD) behavior were observed in DJ-based devices under voltage spikes. By contrast, the performance of RP-phase-based synaptic devices was suboptimal. Results obtained from first-principles density functional theory (DFT) calculations demonstrated that the lateral migration of ions through the organic layer in the RP phase necessitated an energy of 2.79 eV, while the energy required along the surface of the inorganic layer and through the vertical path within the inorganic layer was 0.72 and 0.53 eV, respectively. This significant disparity led to the formation of localized ions, which subsequently generated conductive filaments along the inorganic layer. The rupture of the conductive filaments accounted for the abrupt transition observed in the conductivity state, whereas in the DJ phase, only 0.51 eV was required to pass through the organic layer, a value comparable to the energy needed for passing through the inorganic layer (0.63 and 0.54 eV). The energy required for ions to pass through the organic layer was associated with the van der Waals gap, which was present between the inorganic layers in RP-phase perovskites. However, in DJ-phase perovskites, this gap was effectively eliminated by the formation of two hydrogen bonds between the organic and inorganic layers. Thus, the migration of ions occurred uniformly throughout the entire vertically aligned layer, resulting in a gradual modulation of the width of the depletion layer throughout the region. This broadly applicable approach offers superior linear and symmetric programmability for neuromorphic devices.

#### Electrochemical Metallization Mechanism Type

In contrast to the VCM-type devices, ECM-based devices typically use active metal electrodes. The resistance state change in these devices is primarily attributed to conductive filaments formed via electrochemical redox reactions between the electrode and the perovskite active layer [74]. This mechanism has been verified in the existing literature, where a structure of Ag/PMMA/(Cs_3_Bi_2_I_9_)_0.4_-(CsPbI_3_)_0.6_/Pt structure was conducted [75]. Ag^+^ migration mimics Ca^2+^ influx and electrons migrating through silver conductive filaments mimic neurotransmitter release. Applying a positive bias to the Ag electrode resulted in the production and dissolution of Ag^+^ into the perovskite film. In the presence of an electric field, Ag^+^ migrated toward the bottom Pt electrode, where they underwent reduction and deposition near the electrode surface.

**Fig. 4:**
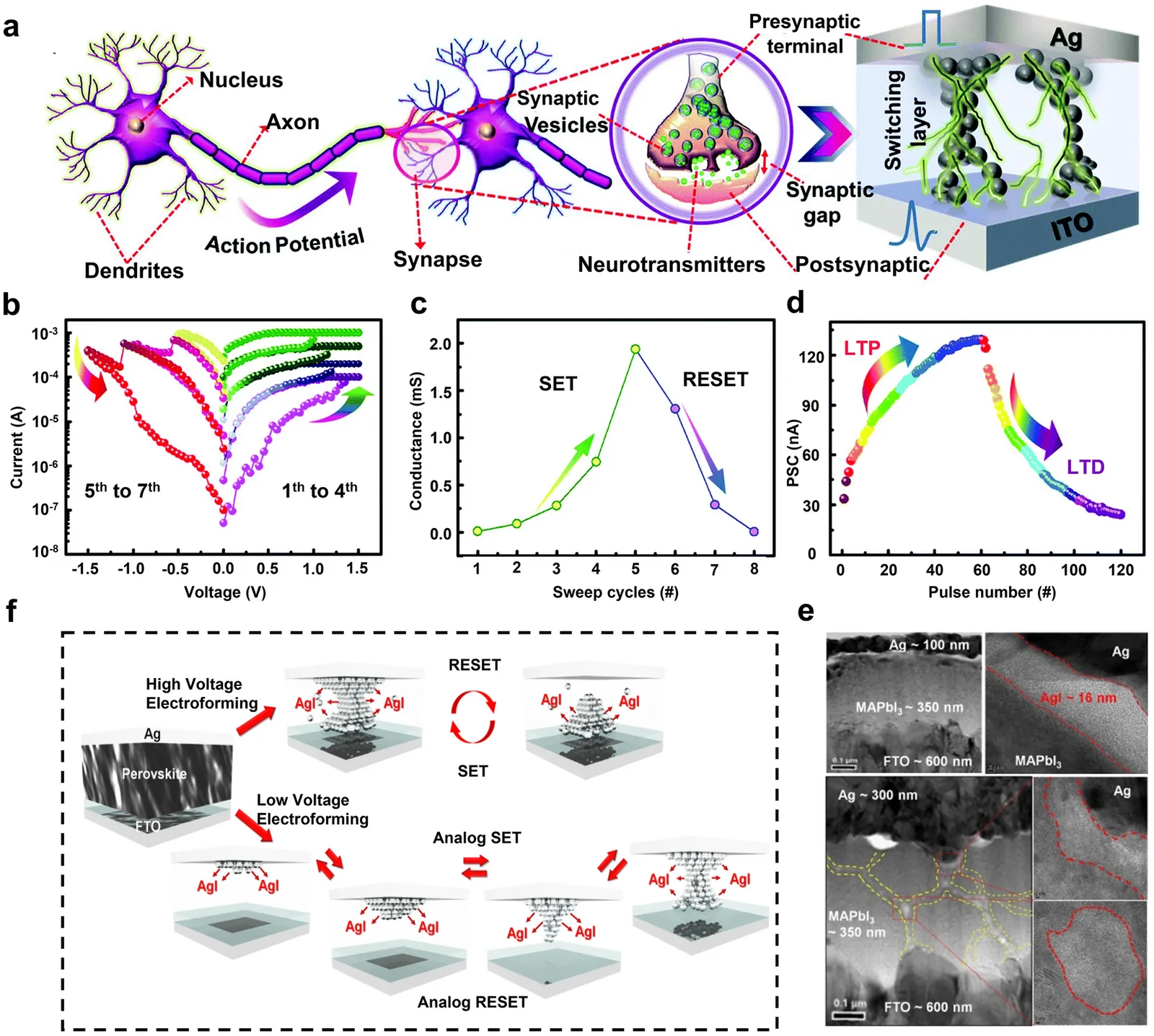
**a** Simulation of biological synapses using Ag/CsSnCl_3_/ITO two-terminal devices. **b, c** Changes in device V_stop_ and conductance during successive positive and negative bias scans. **d** LTP and LTD behavior obtained using 60 positive/negative voltage pulses. Reproduced from Ref. [76] with permission from Royal Society of Chemistry, Copyright 2021. **e** Cross-sectional scanning transmission electron microscopy image of the device. **f** Resistive state switching mechanism of the device. Reproduced from Ref. [77] with permission from Elsevier, Copyright 2020

In ECM-type devices, the migration of halide vacancies can potentially occur concurrently with cation migration. A synaptic device with a memristor configuration has been developed by introducing CsSnCl_3_ film as an active layer, as shown in Fig. 4a [76]. Researchers conducted a comprehensive investigation into the resistive switching behavior. As illustrated in Fig. 4b, c, the V_stop_ value underwent a change during successive positive and negative bias sweeps, increasing from − 0.5 to − 1.5 V. Concurrently, the device conductance also exhibited an increase and subsequent decrease during SET and RESET (from 0.007 to 1.94 mS and then to 0.004 mS). This was attributed to the formation and disruption of silver conductive filaments, as well as the presence of halide vacancies. And it was supported by the relationship between device resistance and both electrode area and temperature. The resistance in the LRS remained constant with increasing device size, indicating that conduction is localized in the conducting filaments. The resistance of the HRS state decreased with increasing temperature, which was attributed to the semiconductor nature of the active layer. On the contrary, the resistance of LRS state showed a tendency to increase. This trend was supported by the fitted resistance–temperature coefficients, which suggested the presence of metal nanowires with diameters on the order of tens of nanometers. It can be inferred that the migration of halide vacancies is not be negligible in the conduction process. The Ag channels were able to traverse the perovskite layer in conjunction with halide vacancies, thereby operating in a synergistic manner within the switching mechanism. While the silver metal filaments serving as the predominant conduit for current flow. Subsequently, the synaptic performance of the device was investigated. When the pulse amplitude was low and the number of the pulse was small, the ions rapidly returned to their original positions, resulting in a sharp increase and rapid decay of conductance. PPF was achieved through the utilization of paired voltage pulses, with a pulse amplitude of 3 V and a pulse width of 10 µs. When a high pulse amplitude or a large number of pulses were applied, a fraction of the ions could move to a position that was far enough from their equilibrium position to make it difficult for them to return to that position. This resulted in a constant current level over time. With 60 consecutive enhancement and suppression pulses (amplitude of ± 1.3 V, pulse width of 100 μs), the device exhibited typical LTP and LTD phenomena (Fig. 4c, d).

To gain a clearer understanding of the resistive switching mechanism, high-resolution transmission electron microscopy was utilized to provide compelling evidence supporting this mechanism in the synaptic device with an Ag/MAPbI_3_/FTO structure [77]. The resistive switching behavior and the realization of synaptic function were thought to be connected with the formation of the AgI phase at the Ag/MAPbI_3_ interface. As illustrated in Fig. 4e, a 16 nm-thick AgI compound can be observed at the interface between the silver and perovskite layers. Additionally, the image depicted the emergence of multiple silver conductive filaments rather than a single filament. In the presence of a substantial positive electroforming voltage, silver atoms migrated into the perovskite layer and form multiple conductive filaments that bridge the top and bottom electrodes. This phenomenon aligned with the abrupt SET and multistage RESET processes. When the voltage amplitude was low, the conductive filaments did not connect the two electrodes but grew gradually. Due to the reaction with iodine, the conductive filaments could not maintain the original shape, which resulted in a decrease in the current level during the SET operation (Fig. 4f). Notably, while static transmission electron microscope (TEM) observation offers a thorough insight into the microstructure of materials, it fails to capture the dynamic changes under external stimulation. Furthermore, the impact of electron diffraction must be taken into account, as it may sometimes lead to misleading conclusions regarding the formation of conductive filaments [78]. To resolve this problem, it is recommended to adopt in situ bias TEM or combine with other characterization methods (e.g., conductive AFM (CAFM) and Kelvin probe force microscopy (KPFM)) as supplements to ensure more reliable and comprehensive results. Specifically, in situ bias TEM enables detailed investigations of filament dynamics, playing a crucial role in real-time clarification of switching dynamics. Such in situ approaches have been utilized in filamentary devices to visualize the real-time growth of conductive filaments (Refs. [79–81]). CAFM allows for the detection of the spatial distribution of local conductivity, which can be readily employed to examine the potential formation and dissolution of local conductive filaments across various resistance states [82]. This applicability has been demonstrated in perovskite memristors to investigate the existence of electric field-induced formation of conductive filaments [83]. KPFM, which can measure the local surface potential, has been employed to distinguish conductive filaments from the surrounding matrix [84].

Overall, the vertical configuration of 2 T perovskite synaptic devices provides outstanding downsizing capacity of cells and high integration density thanks to their simple structure. Additionally, the wide range of options for electrode materials and active layer materials enables multiple operating mechanisms. However, the development of these devices is currently in its nascent stage. An overview of 2 T perovskite synaptic devices, including their key performance metrics, is provided in Table 1.

**Table 1:** Summary of 2 T perovskite synaptic devices

Perovskite		Device structure		Mechanism	Migrating ions	PPF index	Ec	Dynamic	Number of states	Endurance (two-state)	Endurance (LTP + LTD)	Nonlinearity	Retention	References
3D	MAPbI_3_	Au/PVSK/ITO		VCM	A/B/X	–	–	–	–	–	–	–	–	[49]
3D	MAPbBr_3_	ITO/BCCP/PVSK/Al		VCM	X	–	20 fJ*	–	–	–	–	–	–	[87]
3D	MAPbBr_3_	ITO/PEDOT: PSS/PVSK/Bphen/Al		VCM	A/X	192%	–	–	–	–	–	–	–	[72]
3D	MAPbClBr_2_	Al/PVSK/Si		VCM	X	–	5.8pj*	–	–	–	–	–	–	[88]
3D	MA_3_Sb_2_Br_9_	Ag/PMMA/PVSK/ITO		VCM	X/B	–	–	100	25	300	–	–	1 × 10^4^ s	[70]
3D	Cs_3_Sb_2_Br_9_	Au/PVSK/Au		VCM	X	–	–	1 × 10^3^	–	200	–	–	2 × 10^4^ s	[89]
3D	MAPbBr_3_	Au/PVSK/Au		VCM	X	–	14.3fj	–	7	–	186	–	–	[90]
3D	Cs_3_Pb_2_Br_2_I	Ag/P3HT/PVSK/ITO		VCM	X	–	–	10	–	–	–	–	–	[91]
3D	FAPbI_3_	Au/PVSK/ITO		VCM	X	–	–	–	20	–	160	–	–	[50]
3D	Cs_3_Sb_2_I_9_	Al/PVSK/ITO		VCM	X/B	–	–	1 × 10^4^	–	100	–	–	1 × 10^4^ s	[92]
3D	Cs_2_AgBiBr_6_	Ag/PMMA/PVSK/ITO		VCM	B/X	–	188.6 pJ	–	50	110	9	–	–	[93]
3D	CsCu_2_I_3_	Au/PVSK/ITO		VCM	X	–	–	–	50	–	20	1.8/1.3	40 s	[94]
3D	MAPbI_3_	Au/PVSK/ITO		VCM	X	–	13.5aj	–	–	–	–	–	–	[95]
3D	AgBiI_4_	Ag/PMMA/PVSK/FTO		VCM	X	130%		–	–	–	–	–	–	[63]
3D	Cu_2_AgBiI_6_	Ag/PMMA/PVSK/ITO		VCM	X	151%	132 pJ	–	30	–	20	–	–	[64]
3D	Cs_0.05_MA_0.15_FA_0.8_PbI_0.85_Br_0.15_	Ag/Spiro-OMeTAD /PVSK/SnO_2_/ITO		VCM	X	–	–	–	50	–	5	–	–	[85]
3D	δ-FAPbI_3_	Ag/PVSK/ALD-SnO_2_/ITO		VCM	X	–	–	1 × 10^2^	100	1000	10	0.6/3.47	500 s	[96]
3D/2D	(PEA)_2_MA_n-1_Pb_n_Br_3n+1_	Al/PVSK/BCCP/ITO		VCM	X	–	0.7 fJ*	–	–	–	–	–	–	[97]
2D	(PEA)_2_PbBr_4_	Au/PVSK/Graphene		VCM	X	–	400 fJ	10	–	100	–	–	1000 s	[98]
2D	PEA_2_MA_4_Pb_5_I1_6_	Au/PMMA/PVSK/Au		VCM	X	–	–	–	25	–	50	0.07/0.1	–	[99]
2D	Cs_3_Bi_2_I_6_Cl_3_	Al/PVSK/ITO		VCM	X	–	–	1 × 10^4^	25	500	5	–	1 × 10^4^ s	[100]
2D	BA_2_PbI_4_	ITO/PVSK/ITO		VCM	X	126%	0.145 fJ	–	–	–	–	–	–	[101]
2D	BDAMA_n–1_Pb_n_I_3n+1_	Au/PMMA/PVSK/Au		VCM	X	–	2.1pj	–	50	–	50	0.00015/0.0005	1 × 10^4^ s	[73]
1D	(PrPyr)PbI_3_	Ag/PMMA/PVSK		VCM	X	–	–	1 × 10^5^	–	2000	–	–	1 × 10^5^ s	[71]
1D	CsPbI_3_	Ag/PMMA/PVSK/ITO		VCM	X	–	–	1 × 10^2^	50	–	–	–	–	[102]
mixed	Cs_1–x_FA_x_PbBr_3_	Al/V_2_O_5_–y/PVK/PVSK/b–PEI/ZnO/ITO		VCM	A	–	–	–	40	–	5	–	–	[103]
3D	Cs_2_TiBr_6_	Al/PVSK/FTO		VCM	X	–	–	–	100	–	–	–	–	[104]
3D	(Cs_3_Bi_2_I_9_)_0.4_ − (CsPbI_3_)_0.6_	Ag/PMMA/PVSK/Pt		ECM	Ag	–	–	3.2 × 10^8^	–	–	–	–	–	[75]
3D	MAPbI_3_	Ag/MAPbI_3_/FTO		ECM	Ag	–	–	–	–	1000	–	–	1 × 10^4^ s	[77]
3D	FAPbI_3_	Ag/MoO_3_/PVSK/MoO_3_ /ITO		ECM	Ag	–	–	–	–	–	–	–	1 × 10^6^ s	[105]
3D	Cs_3_Cu_2_I_5_	Ag/PMMA/PVSK/ITO		ECM	Ag	–	–	100	20	100	–	–	1 × 10^4^ s	[106]
3D	CsSnCl_3_	Ag/PVSK/ITO		ECM	Ag/X	–	–	100	60	1 × 10^5^	–	–	1 × 10^5^ s	[76]

PPF index is calculated as the ratio of A_2_ to A_1_ (A_2_ divided by A_1_), with A_2_ being the current generated by the second pulse and A_1_ the current elicited by the first pulse

Energy consumption (Ec) is calculated by the product of reading voltage V, output current I, and pulse width t. The value marked with "*" means that Ec was calculated by multiplying the pulse intensity A, output current I, and pulse width t

PVSK, perovskite; BCCP, buffer-capped conducting polymer; ALD, atomic layer deposition; PVK, poly(N-vinylcarbazole)

Regarding modulation methods, it is evident that most synaptic behaviors in these devices are achieved through electrical control, with infrequent utilization of optical pulses. This suggests a deficiency in simulating sensing functions, due to the limited number of stimulus input terminals. Nevertheless, some research teams have explored the realization of photo-assisted synaptic plasticity through lateral 2 T structures featuring active layer exposure [50]. Moreover, the synergistic exploitation of the ion migration barrier and the readout voltage presents an opportunity to realize reconfigurable and optical/electrical controlled perovskite synaptic devices [85].

Concerning the device mechanism types, the resistive switching characteristics are inherently related to the constituent materials but also depend on their interfaces with the electrodes, as well as the structure and properties of these electrodes. The majority of 2 T devices are VCM type, which likely stems from the perovskite’s soft lattice, relatively weak bonds and defect tolerance, leading to easy activation of ions within the perovskite lattice. Concurrently, another contributing factor may be that the use of electrochemically active metal electrodes can lead to unavoidable metal-perovskite electrochemical reactions. Moreover, the competing resistive switching phenomena between the ECM and VCM mechanisms significantly impact the device’s endurance, reliability, and repeatability [86]. It is noteworthy that both filamentary and interfacial mechanisms have been reported. The former is often associated with abrupt conductance states, i.e., digital switching behavior. The latter generally corresponds to multi-level tunable conductance states, i.e., analog switching behavior, which is desirable for synaptic devices and merits further investigation.

In terms of device performance, it can be seen that the most data have been reported for a limited number of switching cycles, with a lack of detailed information on endurance, retention, yield, and variability. Encouragingly, however, low energy consumption, high PPF index, and extremely low nonlinearity have been demonstrated, making these devices worthy of further exploration.

Looking ahead, the correlation between the movement of ions and electrons within the material and the underlying mechanism needs to be further emphasized. The combination of imaging, electronic characterization, and simulation is the recommended approach. Only by fully understanding the mechanism behind the resistive changes can device design and optimization be better targeted toward specific functions and performance parameters.

### Three-Terminal Perovskite Synapses

For three-terminal perovskite synapses, they have the structure of a conventional field effect transistor, consisting of an insulated gate dielectric layer, a semiconductor channel layer, and conductive three electrodes (drain, source, and gate). In conjunction with the exceptional optical properties of perovskites, the presynaptic pulse can be simulated by optical illumination or gate voltage modulation, and the conductance is considered as synaptic weights. Their separated conductance modulation and testing terminals promote enhanced flexibility of synaptic weight modulation [15]. Depending on the operating mechanisms, 3 T perovskite synapses can be further categorized into floating gate (FG) type, heterojunction (HJ) type, defect (DE) type, and ferroelectric (FE) type.

#### Floating Gate Type

**Fig. 5:**
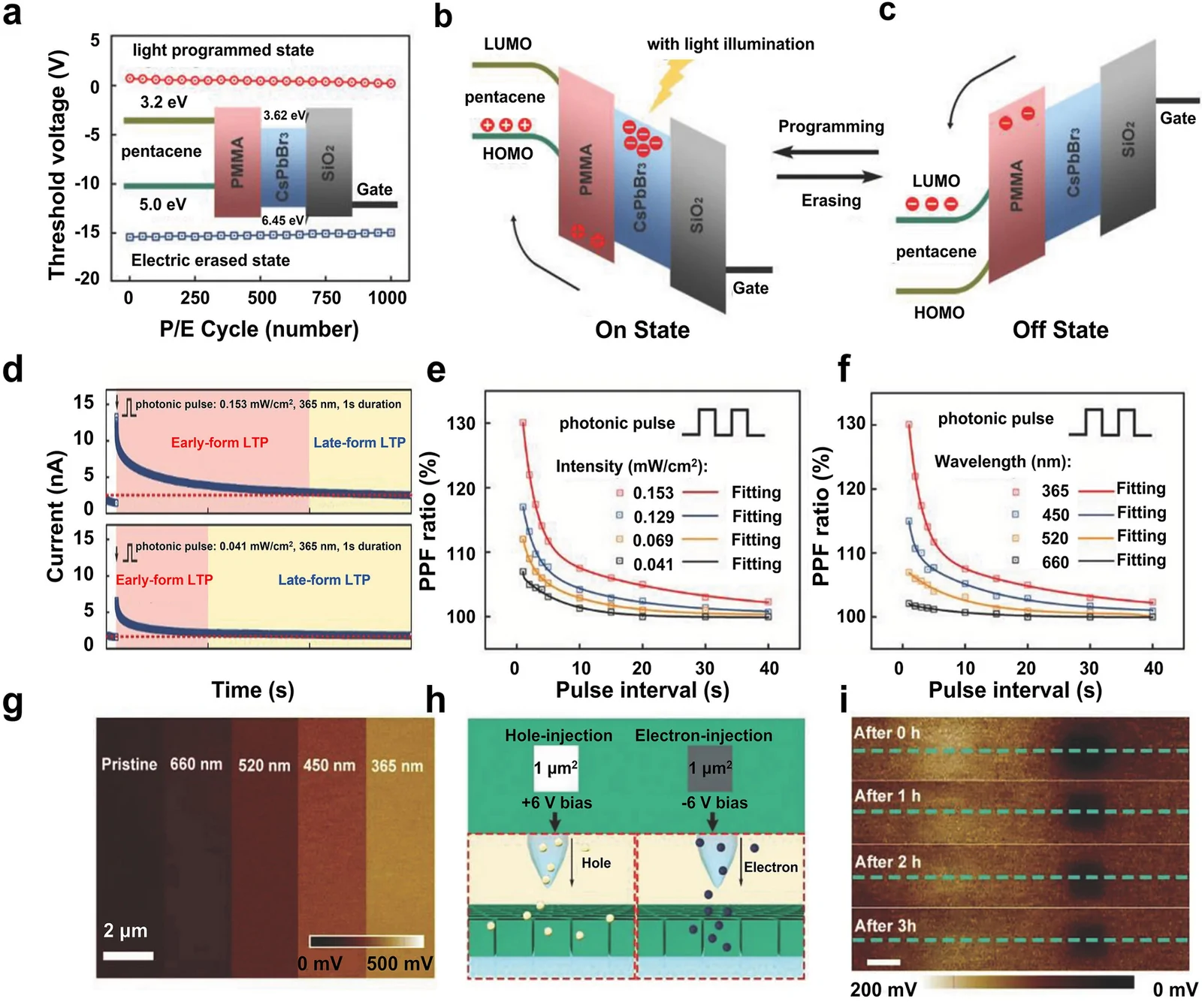
**a** Light-programmed and electric erased state of CsPbBr_3_ QD-based synaptic device. **b, c** Schematic illustration of the mechanism of optical programming and electrical erase operation of the device. **d** EPSC realized with 365 nm photon pulses of different intensities. **e** Simulation of PPF effect by light pulses of different intensities **f** Simulation of PPF effect by light pulses of different wavelengths **g** Surface potential of CsPbBr_3_/PMMA/pentacene films before and after light exposure. **h** Schematic diagram of the process of injecting electrons and holes into the CsPbBr_3_ QDs layer. **i** Snapshots of charge retention properties. Reproduced from Ref. [59] with permission from John Wiley and Sons, Copyright 2018

Perovskite materials typically function as both light-absorbing layers and trapping layers in floating gate-type synaptic devices. Generally, under the stimulation of voltage pulses or light pulses, the majority carriers tunneled into the channel layer, while minority carriers are stored in the floating gate, thereby leading to the generation of non-volatile current [14]. This mechanism has been validated in a synaptic device with a representative structure, specifically Si/SiO_2_/CsPbBr_3_ QDs/PMMA/pentacene/Au structure [59]. As shown in Fig. 5a, after applying the light pulse with a wavelength of 365 nm, an intensity of 0.153 mW cm^−2^, the current of the device shows a state of rising first and then stabilizing, indicating the optical programming operation. The initial state can be restored by applying an electrical pulse with an amplitude of − 50 V, demonstrating an electrical-erasable characteristic. The underlying principles of optical programming and electrical erasure operations are illustrated in the energy band diagrams of Fig. 5b, c. Upon illumination, a substantial number of carriers were generated in the perovskite layer. The photogenerated holes could be readily transferred from the perovskite layer to the channel layer, while the photogenerated electrons were retained in the CsPbBr_3_ conduction band. The remaining electrons act as an additional electric field, further accelerating the holes sweep into the semiconductor channel. The light-induced carrier generation and transfer process in the perovskite layer is thought to be similar to the neurotransmitter inflow process in biological synapses. Light pulse stimulation could induce the production of EPSC. The degree of plasticity varied in direct proportion to the intensity of the light pulse. It was found that the greater intensity of optical stimulus resulted in larger EPSC values and longer decay times (Fig. 5d). PPF index also showed a dependence on light intensity and wavelength shown in Fig. 5e, f. Subsequently, the correlation between synaptic performance and charge carriers was validated through in situ KPFM characterization. The contact potential difference on the pentacene film before and after light irradiation is shown in Fig. 5g. The observed increase in surface potential was attributed to the transfer of photoinduced holes from the perovskite layer to the pentacene layer. And there was a correlation between the surface potential and the wavelength of the incident light, with the potential being greatest at shorter wavelengths. This was in accordance with the pronounced absorption of CsPbBr_3_ in the ultraviolet (UV) region. As illustrated in Fig. 5h, by injecting holes and electrons along the horizontal direction, the dynamic process of charge transport was recorded in a time-accelerated mode. Figure 5i presents several snapshots of the surface potential, where the dark areas represent the hole-trapping state and the bright areas correspond to the electron-trapping state. The faster change in contrast observed in the hole injection region suggests that the holes would drift through the perovskite layer at a faster rate. This electron-trapping property provided compelling evidence for the synaptic characteristics of the device. Therefore, the KPFM findings provided strong support for the conclusion that the synaptic behavior stemmed from the electron-trapping properties of the CsPbBr_3_ QDs layer.

In addition to devices featuring this typical three-layer structure, the floating gate mechanism can also be realized in a simplified two-layer structure. Synaptic devices incorporating a hybrid floating-gate and tunneling layer have also been documented. A synaptic device with a Si/SiO_2_/CsBi_3_I_10_/polyvinyl pyrrolidone (PVP)/Poly[2,5-bis(2-octyldodecyl)pyrrolo[3,4-c]pyrrole-1,4(2H,5H)-dione-3,6-diyl)-alt-(2,2′;5′,2′′′;5′′,2′′′-quaterthiophen-5,5′′′-diyl)] (PDPP4T)/Au structure was demonstrated in which the PVP/CsBi_3_I_10_ hybrid film was prepared by spin-coating to serve the function of inducing and trapping carriers [62].The floating gate structure with PVP allowed for a series of behaviors to be simulated, such as PPF. Interestingly, the decay process of the EPSC was influenced by changes in the operating voltage V_DS_. As the operating voltage increased, the decay process slowed down. Furthermore, modifying the operating voltage of the device could result in a transition from STP to LTP. Moreover, 2D perovskite is a possible choice for floating gate optoelectronic synaptic devices. In a recent study of nonvolatile transistor-based photoresistors, Lai et al. developed a novel organic cation, 4-(5-(2-aminoethyl)thiophen-2-yl)-benzonitrile^+^ (EATPCN^+^) to forming (EATPCN)_2_PbI_4_ [107]. The device exhibited a retention time of greater than 2.4 × 10^5^ s, and remarkable reliability and durability with the on/off ratio remaining at 10^6^ after 10^5^ cycles.

#### Heterojunction Type

Heterojunction-type devices are usually realized through a type-II heterojunction formed by the contact between a carrier transport material and a light-absorbing layer. Under the light irradiation, the heterostructure facilitates the spontaneous separation of photogenerated electron–hole pairs in the light-absorbing layer. Upon the removal of light, the interfacial barriers impede the recombination of carriers, resulting in a delayed decay of the current [108]. This mechanism has been verified in a 3 T synaptic device based on perovskite QDs and regioregular poly (3-hexylthiophene-2, 5-diyl) (commonly referred to as P3HT) [109]. The hybrid heterojunction layer offered potential for streamlining the device architecture, which facilitated interfacial contact between P3HT and perovskite, ultimately achieving efficient charge carrier separation. The band alignment between the charge transport layer and the charge capture layer played a crucial role in determining the storage behavior. Upon light irradiation, the energy level offset between P3HT and perovskite induced exciton dissociation. Holes were then transferred to P3HT, while electrons were stored within the perovskite. The application of a negative V_GS_ facilitated the re-injection of holes in P3HT into the perovskite, thereby inducing hole–electron recombination and restoring the device conductance to its initial state. Time-resolved photoluminescence (TRPL) results showed a significant reduction in the average fluorescence decay time after mixing perovskite with P3HT, which confirmed the transfer of photogenerated carrier between the two materials.

It is worth noting that HJ-type devices exhibit impressive synaptic functions, particularly in terms of energy consumption and PPF index. In an optoelectronic synaptic device based on poly (d-decanolactone)-based conjugated block copolymer and perovskite quantum dots, it was proposed that the proper self-assembly of P3HT facilitated the self-aggregation of perovskite quantum dots [110]. The heterogeneous interface between the two was thus increased, resulting in enhanced charge dissociation and improved transport barriers. Consequently, in terms of energy consumption, under a voltage of − 0.1 mV and a light duration of 1 ms, the device exhibited an ultralow energy consumption of 0.3 aJ, which is the lowest reported value for a similar device to date. Wang et al. employed a surface energy-induced strategy to prepare hybrid films consisting of perovskite dots distributed in elastomeric SEBS in a quasi-continuous microsphere morphology [111]. The quasi-continuous microsphere morphology of perovskite provided good heterogeneous contacts and sufficient phototropic carrier transport. A PPF index of up to 270% was attained at time interval (Δt) of 0.5 s.

#### Defect Type

Operating mechanism of defect-type devices is linked to the defect-induced charge trapping and de-trapping process [112]. The defects focused on in this section are confined to those within the perovskite film and at its interface with non-charge transport materials. They capture the electrons or holes generated by light and release these charge carriers upon the application of a voltage stimulus. During this process, the device’s resistance is modulated.

**Fig. 6:**
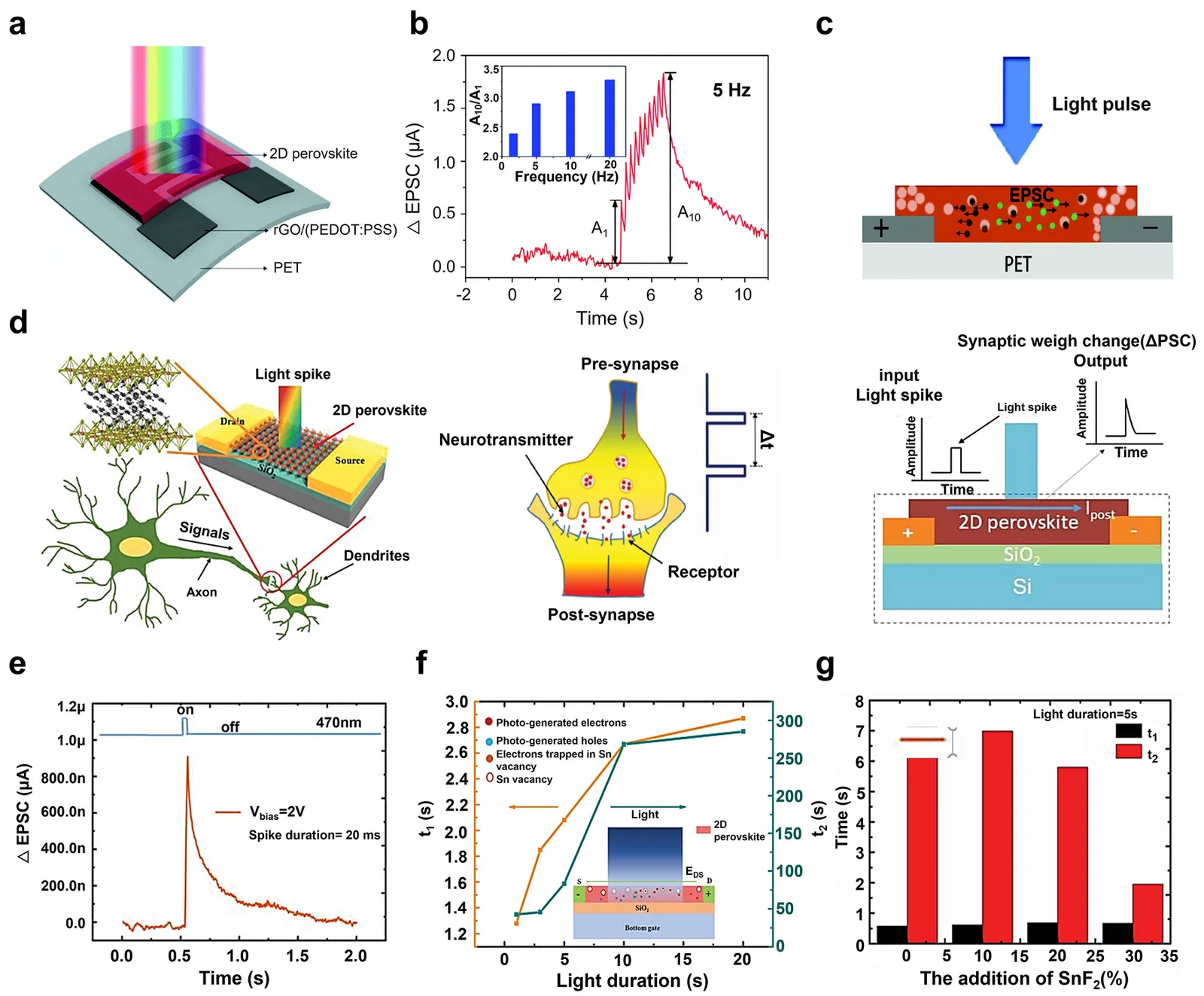
**a** Flexible perovskite two-terminal synaptic device. **b** ΔEPSC under 10 light pulses. **c** Scheme of the working mechanism of the synaptic device (white, black, and green balls correspond to vacancies, electrons, and holes, respectively). Reproduced from Ref [113]. with permission from Royal Society of Chemistry, Copyright 2018. **d** Schematic of a 3 T perovskite synaptic device and a biological synapse. **e** EPSC triggered by light pulses. **f** Plot of time constant as a function of light duration. **g** Effect of SnF_2_ addition concentration on time constants t_1_ and t_2_. Reproduced from Ref. [114] with permission from John Wiley and Sons, Copyright 2019

Defect-type synaptic devices initially drew inspiration from that observed in two-terminal synaptic devices. Utilizing inherent defects in perovskite materials, a synaptic device on PET based on two-dimensional perovskite (PEA)_2_SnI_4_ has been developed, as illustrated in Fig. 6a [113]. As shown in Fig. 6b, a gradual increase in EPSC was observed when ten light pulses were applied to the device. It was hypothesized that this phenomenon was associated with the trapping of carriers by Sn vacancies. The light pulse generated free carriers, while some electrons could be trapped by the aforementioned-vacancies. The electrons that were trapped could result in an increase in the number of holes produced by the second pulse (Fig. 6c). In a manner analogous to the materials utilized in the aforementioned study, a 3 T perovskite synaptic device based on (PEA)_2_SnI_4_ has been further demonstrated as shown in Fig. 6d [114]. The generation of excitatory postsynaptic currents was initiated at a 2V bias voltage through the application of a light pulse with a wavelength of 470 nm (duration of 20 ms, intensity of 11.6 µW cm^−2^) (Fig. 6e). By varying the frequency of incident light or employing repetitive light stimulation, the synaptic device was able to successfully simulate the transition from STP to LTP. Also, it was found that as the light duration increased, photoresponsivity rose, accompanied by an extended decay time. A two-exponential time model (t_1_ and t_2_), which assumed a shorter and longer time constant, respectively, was employed to fit the light duration data. The time constants t_1_ and t_2_ increased with the duration of illumination and became saturated when the duration exceeds 10 s. The authors proposed that these time constants could be attributed to the relaxation of two distinct types of trapped states within the perovskite material. The t_1_ was associated with shallower traps, while t_2_ was linked to deeper traps. For STP under short pulse durations, the capture and release of photogenerated carriers was relatively brief, resulting in a rapid decay of channel conductance. When the light intensity was sufficiently high, the carriers might be captured by deeper traps. Trapped electrons might continue to induce holes through the photogating effect, resulting in longer de-trapping time. The origin of traps may be attributed to shallow traps, which were generally vacancy defects, uncompensated dangling bonds or structural defects presented in synthesized 2D materials. Deeper traps were attributed to Sn vacancies, which were positively charged and could trap photogenerated electrons, as illustrated in the inset in Fig. 6f. To substantiate this hypothesis, the hole inhibitor SnF_2_ was introduced, which has the capacity to mitigate the oxidation of Sn^2+^ to Sn^4+^. It was observed that t_1_ showed a slight change while t_2_ experienced a significant decrease as the SnF_2_ concentration increased. This indicated that the photogating effect caused by the reduction of Sn vacancies was suppressed (Fig. 6g). The X-ray photoelectron spectroscopy results quantitatively indicated the amount of Sn^4+^, providing strong support for this conclusion. The Sn^4+^ content in the SnF_2_-containing perovskite film was significantly lower than in the pristine film, demonstrating the trapping effect of Sn vacancies.

#### Ferroelectric Type

**Fig. 7:**
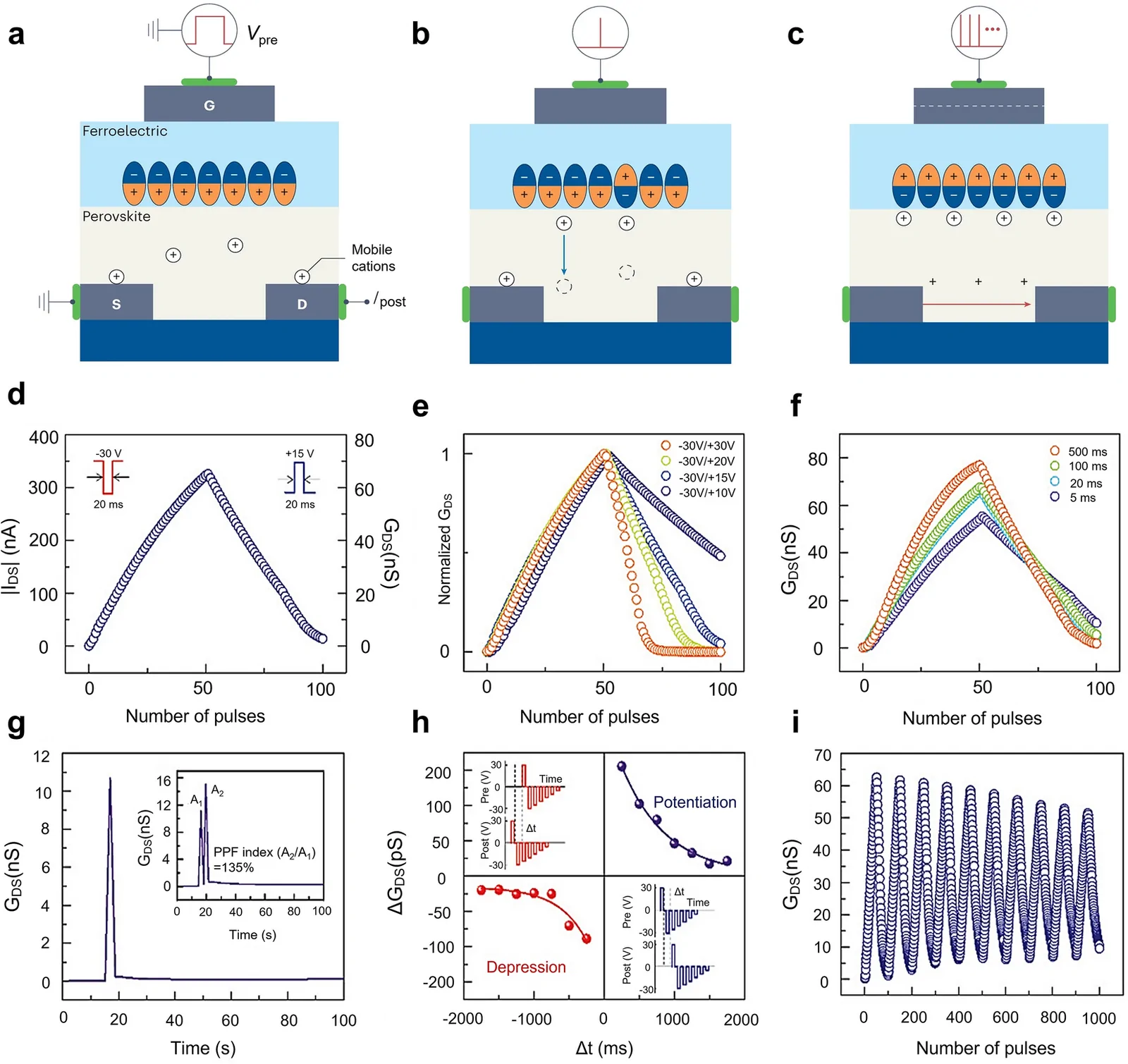
**a–c** Schematic diagram of the operation of a ferroelectric perovskite synaptic device. Reproduced from Ref. [24]. with permission from Springer Nature, Copyright 2023. **d** LTP and LTD characteristics of the device. **e** LTP and LTD characteristics for different pulse amplitudes. **f** LTP and LTD characteristics for different pulse widths. **g** Conductance updates under 1000 repetitions of stimulation. **h** PPF behavior. **i** STDP behavior as a function of Δt. Reproduced from Ref. [116]. with permission from John Wiley and Sons, Copyright 2021

The key to ferroelectric-type devices lies in the use of ferroelectric materials to replace traditional dielectric layers, which allows for the maintenance of updated channel conductance via the spontaneous electrode polarization of the materials [115]. Taking advantage of the unique semiconductor-ionic properties of perovskite materials, a perovskite synaptic device comprising a CsPbBr_3_ channel layer and a ferroelectric material poly(vinylidene fluoride-ran-trifluoroethylene) (P(VDF-TrFE)) dielectric layer was constructed [116]. The working principle of synaptic plasticity realization is illustrated in Fig. 7a–c. When the ferroelectric dielectric layer exhibited a downward residual polarization, the movement of cations (red circles) in the perovskite channel layer impeded the transport of carriers, resulting in a reduction in channel conductance. When a negative bias was applied to the gate, the ferroelectric layer was partially polarized. Cations were attracted by the ferroelectric polarization, and the channel conductance increased. Due to the repulsive forces generated by the predominantly downward polarization, most of the cations diffused back to their original positions, resulting in a rapid decay in channel conductance, thereby simulating simulated STP. Upon the application of a long pulse sequence, ferroelectric polarization was reversed. A high level of conductivity was obtained since the mobile cations were depleted, thus mimicking the transition from the STP to the LTP. The width and intensity of the pulse were critical to the linearity of the LTP and LTD. As shown in Fig. 7d, LTP and LTD were obtained with good linearity using pulses of − 30/ + 15 V, respectively, with a pulse duration of 20 ms. For LTD, too large voltage pulses (30, 20 V) led to abrupt changes in the conductance state, as shown in Fig. 7e. In addition, the pulse width also played an important role (Fig. 7f). Pulses duration exceeding 100 ms led to fast saturation and early suppression, whereas a short pulse width of 5 ms could not completely reverse the polarization. The application of a series of pulses resulted in the gradual polarization of the ferroelectric material. As a consequence, the device was capable of mimicking the PPF behavior, with a PPF index of 135% (Fig. 7g). The spike-timing-dependent plasticity (STDP) learning rule was simulated by applying pre- and post-neuronal spikes to the gate and drain, respectively, and varying the Δt between them (Fig. 7h). As illustrated in Fig. 7i, the device was capable of withstanding up to 1000 repetitions of switching.

In summary, the relatively complex structure of 3 T synaptic devices supports independent signal transmission and weight modulation channels, offering minimal crosstalk, and parallel processing capabilities. Additionally, the flexibility in selecting each functional layer facilitates the diversity of operating mechanisms. Critical metrics of 3 T perovskite synapses are summarized in Tables 2 and 3.

**Table 2:** Summary of 3 T floating gate (FG)-type, defect (DE)-type, and ferroelectric (FE)-type perovskite synapses

Perovskite		Device structure	Mechanism	Wavelength (nm)	PPF index	Ec	Dynamic range	Number of states	Endurance (two-state)	Endurance (LTP + LTD)	Nonlinearity	Retention	References
3D	CsBi_3_I_10_	Si/SiO_2_/PVSK + PVP/PDPP4T/Au	FG	430	–	–	1 × 10^3^	50	–	–	–	1.4 × 10^3^ s	[62]
2D	MAPbBr_3_	Si/SiO_2_/PVSK/PS/pentacene/Au	FG	405, 530	–	5.8 pJ	–	50	–	50	–	2 × 10^3^ s	[118]
2D	(EATPCN)_2_PbI_4_	Si/SiO_2_/PVSK/pentacene/Au	FG	365	–	1.12fj	1 × 10^6^	250	1 × 10^5^	5	0.92/2.58	2.4 × 10^5^ s	[107]
0D	CsPbBr_3_	Si/SiO_2_/PVSK/PMMA/ pentacene/Au	FG	365, 450, 520, 660	130%	1.4 nJ	1.86 × 10^5^	20	1000	–	–	3000 s	[59]
0D	CsPbBr_3_	Si/SiO_2_/PVSK/PVP/PVDT–10/Au	FG	400, 500, 650	151%	4.1pj	–	–	–	–	–	–	[119]
0D	Cs_2_AgBiBr_6_	Si/SiO_2_/PVSK/PMMA/PVDT–10/Cr/Au	FG	330, 430, 470, 640	168%	–	–	30	–	–	–	–	[120]
0D	CsPbBr_3_	Si/SiO_2_/PVSK/h–BN/graphene/Au	FG	520	196%	–	–	100	–	–	–	–	[121]
0D	Cs_3_Bi_2_I_9_	PET/Al_2_O_3_/PVSK + PMMA/DPPDTT/Au	FG	405, 532, 635	–	–	–	200	–	–	–	–	[122]
0D	Cs_2_AgBiBr_6_	Si/SiO_2_/PVSK/OS /IDTBT/Au	FG	365, 430, 480, 600	–	–	1 × 10^3^	50	–	–	–	5 × 10^3^ s	[123]
0D	Zr–CsPbI_3_	Si/SiO_2_/PVSK/PMMA/ pentacene/Au	FG	405, 520, 650	–	–	1 × 10^2^	50	2500	8	–	300 s	[124]
2D	(PEA)_2_SnI_4_	Si/SiO_2_/PVSK/Au	DE	470	129.7%	–	–	–	–	–	–	–	[114]
0D	CsPbBr_3_	Si/P(VDF–TrFE)/PVSK/Au	FE	–	135%	–	1.9 × 10^3^	50	70	10	–	1 × 10^4^ s	[116]

IDTBT, indacenodithiophene-co-benzothiadiazole

**Table 3:** Summary of 3 T heterojunction (HJ)-type perovskite synapses

Perovskite		Device structure	Mechanism	Wavelength (nm)	PPF index	Ec	Dynamic range	Number of states t/b	Endurance (two-state)	Endurance (LTP + LTD)	Retention	References
3D	CsBi_3_I_10_	Si/AlO_x_/sc–SWCNT/PVSK/Au	HJ	500	–	–	5 × 10^6^	100	–	–	–	[125]
3D	MAPbI_3_	Si/SiO_2_/Si NM/PVSK/PMMA/Au	HJ	532	–	1pj	–	–	–	–	–	[126]
3D	Cs_2_AgBiBr_6_	Si/SiO_2_/IGZO/PVSK/Au	HJ	365, 455, 505, 660	206.90%	13 nJ	–	–	600	–	–	[127]
2D/3D	FAPbI_3_	Li–AlO_x_/InO_x_/PVSK/ZnO	HJ	465, 525, 625	145%	36.77 nJ	6 × 10^5^	–	–	–	–	[128]
2D	BA_2_PbBr_4_	Si/SiO_2_/IZTO/PVSK/Au	HJ	365, 460, 530, 660	–	–	1 × 10^4^	–	50	–	–	[129]
2D	PEA_2_SnI_4_	ITO/PVP/PVSK/Y6/Au	HJ	450, 520, 650, 808	–	–	–	–	–	–	–	[130]
1D	CsPbBrI_2_	Si/SiO_2_/PVSK/PDVT–10/Au	HJ	365, 450, 515, 635, 735	–	–	–	–	–	–	–	[131]
1D	(α–MBA)PbI_3_	Si/SiO_2_/SWCNT/PVSK/Au	HJ	340,375, 395, 430, 447	230%	–	–	–	–	–	–	[132]
0D	CsPbBr_3_	Si/SiO_2_/PVSK/PQT–12/Au	HJ	500	–	–	–	–	–	–	–	[133]
0D	CsPbBr_3_	Si/polymer ion gel/MoS_2_/PVSK/Au	HJ	405	–	42.93 nJ	–	–	–	–	–	[134]
0D	CsPbBr_3_	Si/SiO_2_/PVSK/DPPDTT/Au	HJ	450	170%	0.5 fJ	–	–	50	–	–	[135]
0D	CsPbBr_3_	Si/SiO_2_/MoS_2_/PVSK/Au	HJ	405	–	–	1 × 10^4^	–	–	–	–	[136]
0D	MAPbBr_3_	Si/SiO_2_/graphene/PVSK/Au	HJ	440	–	–	–	–	20	–	–	[137]
0D	CsPbBr_3_	Si/SiO_2_/IGZO/PVSK/PMMA/ITO	HJ	445, 525	140%	–	20	–	20	10	–	[138]
0D	CsPbBr_3_	Si/SiO_2_/PVSK/TIPS/Au	HJ	450	160%	76 fJ	–	–	–	5	–	[139]
0D	CsPbBr_3_	Si/SiO_2_/IGZO/PVSK/IGZO/Au	HJ	650	–	130 pJ	–	–	–	–	–	[140]
0D	FAPbBr_3_	Si/SiO_2_/SWCNT/PVSK/Au	HJ	405, 532	–	75 fJ	–	–	–	–	–	[141]
0D	CsPbBr_3_	Si/SiO_2_/PVSK/P3HT/Au	HJ	405, 450	160%	0.18 fJ	–	–	–	–	5 × 10^3^ s	[142]
0D	FAPbBr_3_	Si/SiO_2_/PVSK/P3HT/Au	HJ	450	–	30 aJ	–	10	–	–	1 × 10^4^ s	[109]
0D	CsPbBr_3_	Si/SiO_2_/PVSK/PS /C8–BTBT/Au	HJ	365, 450, 500, 600	–	0.11 fJ	–	100	50	–	–	[61]
0D	CsPbBr_3_	Si/Al_2_O_3_/CNT/PVSK/Au	HJ	405	180%	–	–	–	–	–	–	[143]
0D	CsPbBr_3_	Si/SiO_2_/SWCNT /PVSK/PDTT4T /PEDOT: PSS	HJ	450	–	1.3 fJ	–	–	–	–	–	[144]
0D	CsPbBr_3_	Si/SiO_2_/IGZO/PVSK/IGZO/Mo	HJ	520	–	–	–	–	–	–	–	[145]
0D	CsPbBr_3_	Au/ICCN/PVSK/DPPDTT/Au	HJ	450	–	0.4 pJ	–	20	150	10	600 s	[146]
0D	CsPbBr_3_	Si/SiO_2_/PDPP–TT/PVSK/Au	HJ	365	–	–	–	–	–	–	–	[147]
0D	CsPbBr_3_	Si/SiO_2_/PVSK/PDL–b–P3HT–b–PDL/Au	HJ	450, 530, 650	198%	0.3 aJ	4.9 × 10^5^	–	–	–	–	[110]
0D	CsPbBr_3_	Si/SiO_2_/PVSK/DPPDTT/Au	HJ	450	–	27.9 aJ	3.02 × 10^7^	50	–	–	–	[148]
0D	CsPbBr_3_	SEBS/CNTs/DPPDTT + SEBS/PVSK	HJ	365, 460, 520, 625, 808	270%	15 aJ	–	–	–	–	–	[111]

TAPC, 4,4′-Cyclohexylidenebis[N,N-bis(4-methylphenyl)benzenamine]; sc-SWCNT, semiconducting single-walled carbon nanotubes; IZTO, indium zinc tin oxide; PQT-12, poly(3,3-didodecylquarterthiophene); IGZO, indium gallium zinc oxide; PDL, poly(δ-decanolactone); CNTs, carbon nano tubes; SEBS, styrene ethylene butylene styrene.

Regarding modulation method, it is evident that most synaptic behaviors in these devices are executed through the synergistic regulation of light and electric. This modulation strategy enables 3 T perovskite synapses to integrate spatiotemporal information processing ability, thereby supporting neuromorphic vision computing applications. Generally, floating gate, heterojunction, and defect-type synaptic devices are based on photo-programming and electrical erasing. Ferroelectric-type devices are distinct, less frequently reported, and typically utilize electrical stimulation for programming, though researchers have developed a novel opto-ferroelectric synapse [117].

In terms of performance and functionality, 3 T perovskite synapses demonstrate superior performance across several key metrics, such as energy consumption and dynamic range. In the future, device durability and retention merit further improvement through material and structural innovation. Significantly, research on perovskite optoelectronic synaptic devices is almost entirely predicated on the photoconductive effect, characterized by a photocurrent greater than the baseline dark current. However, this constrains the applicability of perovskite synaptic devices in certain specific scenarios. One key challenge lies in developing a universal strategy to achieve negative photoconductivity (where the baseline dark current is higher than the current under illumination) to achieve all optical-controlled synaptic devices. This would effectively simulate inhibitory synapses, addressing a lack of integrity in synaptic function simulation and filling gaps in specific application scenarios. To achieve this characteristic, modulating the number of charge carriers can directly reduce the photocurrent, and slowing down carrier movement is also a feasible approach. The switching between positive and negative photoconductivity can be modulated by varying the light wavelength, intensity, the polarity of the applied bias, and the design of device structure [23].

### Stability of Perovskite Synapses

Although significant advancements have been made in improving performance parameters such as energy consumption and linearity of perovskite synaptic devices, long-term device stability and reliability has been the most pressing issue for both 2 T and 3 T emerging perovskite synapses. Some reports on record devices rarely include stability measurements regarding endurance, stability, and yield of the devices. Such reliability challenges at the device level are generally dependent on the memristive material. The inherent sensitivity of perovskite materials to environmental factors such as humidity, temperature, and light leads to the degradation of synaptic device performance over time. In this section, the environment-induced decomposition mechanisms of halide perovskites are elucidated. Meanwhile, the typical strategies for overcoming these stability challenges are summarized.

Degradation upon water exposure is a major factor affecting perovskite stability, compromising the long-term stability of synaptic devices [149]. Taking the typical MAPbI_3_ as an example, it can easily decompose into PbI_2_, CH_3_NH_2_, HI, and other products upon reaction with water, with the reaction process being irreversible [150]. In addition to the degradation processes, surface defects and grain boundaries are generally regarded as degradation initiators in perovskite films, providing pathways for water molecule penetration [151]. Temperature also serves as an important factor that induces the degradation of halide perovskites, an essential aspect in evaluating halide perovskites properties is their thermal stability [152]. This thermal instability stems from the polycrystalline nature of perovskite materials and the volatility of organic cations [153]. At low temperatures, MAPbI_3_ tends to decompose into PbI_2_, CH_3_NH_2_, and HI, while at high temperatures, it prefers to decompose into NH_3_, CH_3_I, and PbI_2_ [154]. In comparison, all-inorganic halide perovskites exhibit superior thermal stability [155]. For perovskite synaptic devices, the impact of light demands thorough exploration, as these devices need to undergo repeated optical programming and erasure cycles. Even without other environmental stimuli, light can trigger perovskite degradation. Particularly, UV and blue wavelengths are especially impactful due to their large photon energy [156]. In the presence of oxygen, perovskites undergo rapid decomposition under light irradiation [157]. PbI_2_ films degrade into metallic Pb^0^ and I_2_ gas, while the N–H bonds in perovskites also dissociate upon exposure to light [149].

In addition to the aforementioned environmental factors, electric field-induced degradation is also of great consequence as its working condition. Applied bias may reduce the content of Pb^2+^ and I^−^ in MAPbI_3_ with an uneven distribution and can also trigger the irreversible degradation into yellow PbI_2_ [158, 159]. Moreover, excessive ion migration induced by the electric field can generate defect clusters near the interface, which impairs the stability of conductive filament formation and ultimately affects device variation [160].

The stability of perovskite materials is also affected by interfacial reactions with metal electrodes [149]. Common metal electrodes, such as Ag and gold Au, are susceptible to corrosion when in contact with perovskites. On the one hand, corrosion stems from halide species like I⁻ migrating out of the perovskite layer which result in reactions between metal and halide ions, reducing conductivity over time. On the other hand, the metal ions generated by corrosion are capable of diffusing into the perovskites, further promoting its deterioration [161]. Reports indicate that almost all interactions between metal electrodes and perovskites lead to irreversible declines in performance and stability [162].

Put simply, under different external environments and operating conditions, both the intrinsic instability of perovskites themselves and the instability at interlayer interfaces are crucial factors affecting the optical/electrical response performance and operational stability (including retention and endurance) of biomimetic synaptic devices. To date, with respect to the stability requirements of biomimetic synapses, several strategies can be employed to stabilize the halide perovskites in biomimetic synapses, include composition engineering, additive engineering, and barrier layer. Composition engineering leverages chemical substitution or alloying at the A, B, and X lattice sites to enhance perovskite stability [163]. For A-site alloying, common candidates such as MA⁺, FA⁺, Cs⁺, and Rb⁺ can be incorporated into the perovskite lattice individually or in a mixture to improve material stability [152]. The introduction of large-radius cations (e.g., PEA⁺ or BA⁺) represents another effective approach, as these leads to the collapse of the 3D perovskite crystal structure, forming a quantum-confined, usually 2D structure [149]. The presence of large, hydrophobic cation layers could boost the stability of perovskite films [164]. For B-site alloying, replacement of Pb^2+^ with non-toxic dopants (e.g., Sn^2^⁺, Bi^3^⁺) not only enhances stability but also mitigates concerns regarding the toxicity of Pb-based halide perovskites [165]. X-site alloying involves various combinations of Cl⁻, Br⁻, and I⁻ ions, which directly modulate the band edge, tune the band gap, regulate grain growth, and improve the chemical stability [149]. Additive engineering enables the regulation of crystallization to increase grain size and passivate grain boundaries [166]. Reduced defect density at the surface and grain boundaries can suppress degradation and ion migration, thereby enhancing stability [167]. A variety of additives, including organic ammonium halide salts, ionic liquids, and organic additives, have been demonstrated to improve the moisture-/oxygen-/light-stability of perovskites [45, 168–171]. Introducing a barrier layer at the interface can enhance the stability of perovskites through preventing the intrusion of environmental factors, eliminating interface defects, blocking ion migration, and inhibiting interface chemical reactions [149]. Specifically, buffer layer, especially polymer materials, can be introduced at the perovskite/air interface to isolate moisture or oxygen and prevent perovskite degradation [162]. Alternatively, 2D materials, metal oxides, and functional polymers can be used as interface layers between perovskite and metal electrode or charge transport materials, to passivate surface defects and prevent ion migration [172–174].

Despite being a challenging and persistent problem in perovskite synapses, the operational stability is maturing by using many enhancing techniques. Building on the ongoing intensive efforts in this field, we anticipate that highly stable perovskite biomimetic synapses modules are achievable.

## Perovskite Synapses for Neuromorphic Vision Computing

In contrast to traditional vision computing architecture based on the von Neumann architecture, neuromorphic vision computing represents an approach that mimics the human visual system to perceive, store, and pre-process optical information in an energy-efficient manner [175]. Perovskite synapses possess considerable superiority in neuromorphic vision computing owing to their customizable synaptic plasticity and compatible manufacturing processes. Perovskite electronic synapses enable neuromorphic vision computing by accelerating vector multiplication via their multi-level conductance states, thus eliminating bandwidth limitations and reducing data movement. Perovskite optoelectronic synapses with space and time-dependent plasticity can directly integrate visual processing within each device, offering a potential approach to in-sensor neuromorphic vision computing [176]. In this chapter, perovskite synaptic devices applied to neuromorphic vision computing are introduced, with a focus on the manifestation of their synaptic plasticity in specific application scenarios.

### Optical Information Preprocessing

**Fig. 8:**
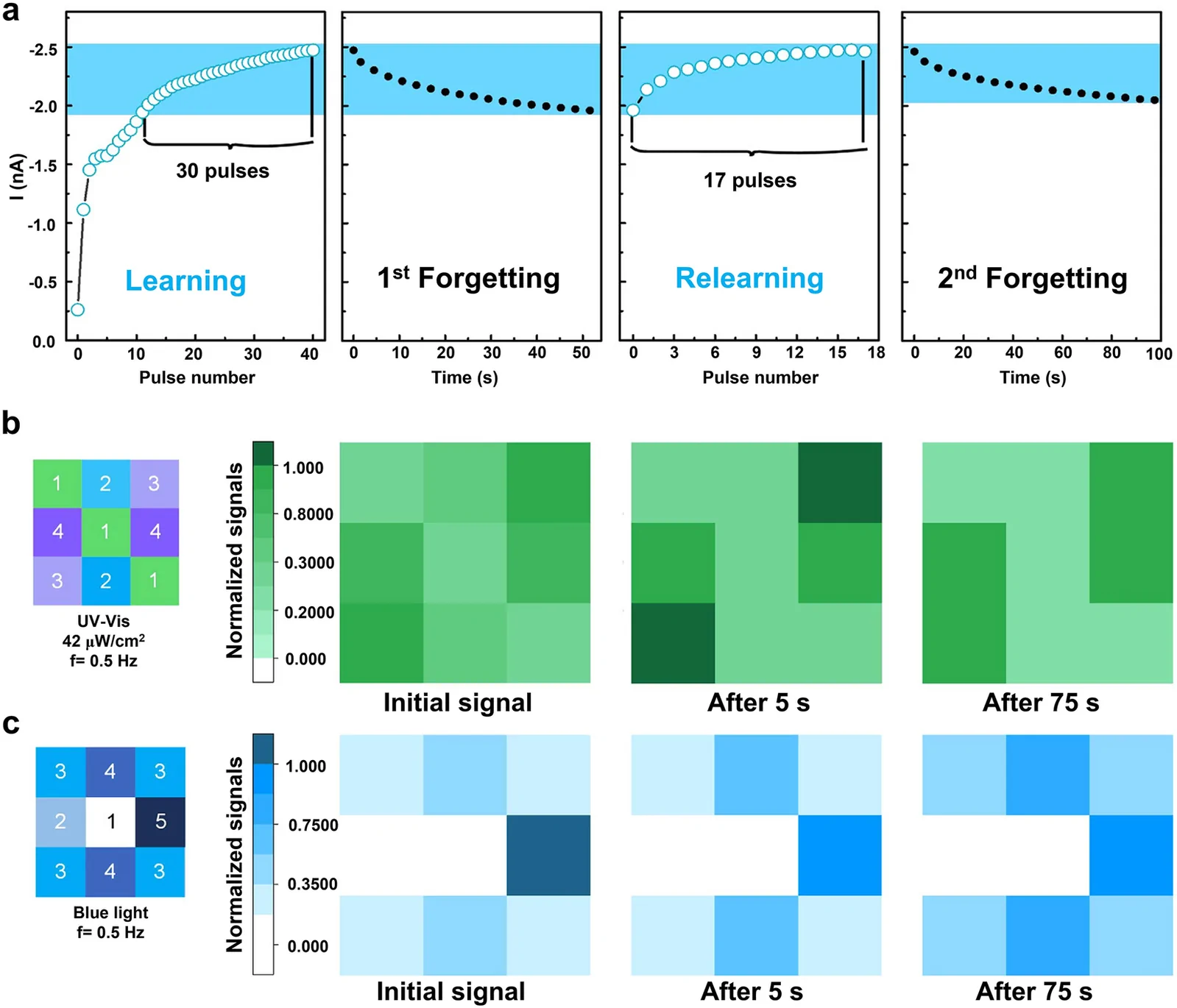
**a** Learning-forgetting-relearning-forgetting process. **b, c** Effect of light wavelength and light intensity on memory. Reproduced from Ref. [139] with permission from American Chemical Society, Copyright 2021

As optoelectronic devices with excellent photosensitivity, perovskite synapses possess the ability to perform preliminary processing of optical information, thereby laying the foundation for subsequent recognition processes. Fundamentally, by leveraging the PPF behavior, perovskite synapses can achieve image contrast enhancement function, a process that also resembles the human learning process. A CsPbBr_3_/6,13-bis (triisopropylsilylethynyl)-pentacene (TIPS) phototransistor wherein CsPbBr_3_ exhibited an island-like structure was successfully fabricated [139]. The process of learning, forgetting, relearning, and forgetting has been successfully simulated. As illustrated in Fig. 8a, following the stimulation of the channel with 40 light pulses, the EPSC exhibited a notable enhancement and subsequently decayed to a specific level after 100 s. Subsequently, the application of only 17 light pulses resulted in the restoration of the EPSC to its initial level, with the current level after 100 s exhibiting an increase. This represents an identical mnemonic process observed in the human brain, whereby the learning curve is reduced and memory retention is enhanced. Furthermore, color can also elicit the distinctive effect of human memory. Different colors have been demonstrated to exert a specific influence on human memory; this behavior has also been verified in the device. As shown in Fig. 8b, the positions 1, 2, 3, and 4 represented distinct light wavelengths, specifically 365, 400, 450, and 500 nm, respectively. It can be observed that the memory evoked by blue light stimulation was more robust and exhibited a slower decline. Furthermore, the light intensity exerts a comparable influence on the memory properties. The positions indicated as 1, 2, 3, 4, and 5 in the image correspond to light intensities that range from low to high. The memory effect induced by stronger light pulses was more pronounced (Fig. 8c). Furthermore, image contrast was observed to be enhanced, a result of significant practical value in neuromorphic vision systems.

**Fig. 9:**
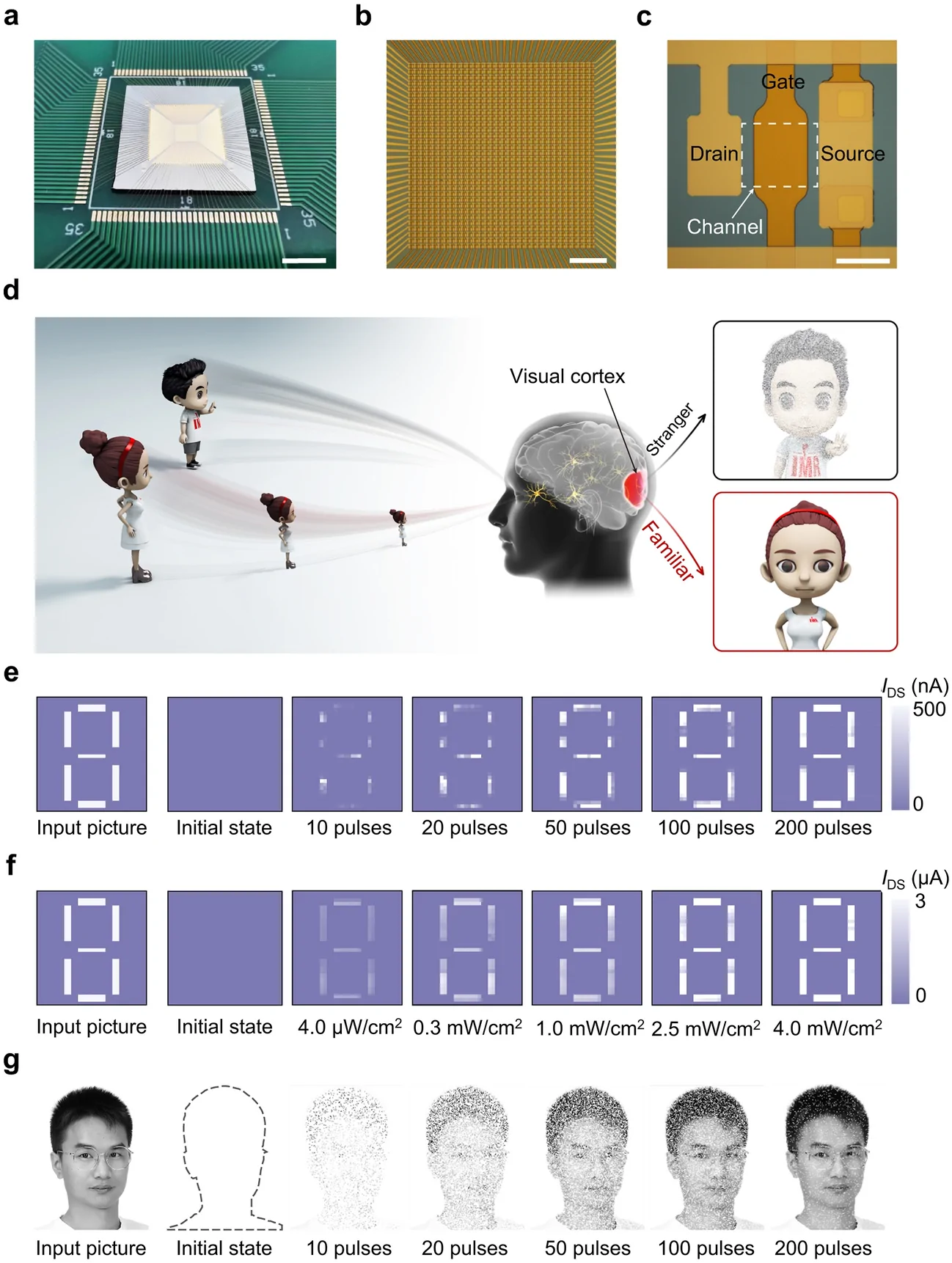
**a** Synaptic array chip bonded with PCB. **b** Optical micrograph of a 32 × 32 synaptic device array. **c** Magnetic image of a single synaptic transmission device. **d** A brief illustration of the facial feature memory of human. **e, f** Weight results of a number-8 pattern after training pulses with different numbers and light intensity. **g** Simulation of a person’s facial features in the initial state and after training. Reproduced from Ref. [143] with permission from Springer Nature, Copyright 2021

The utilization of larger arrays comprising more synaptic devices can facilitate the discernment of finer image details. Zhu et al. fabricated heterojunction-type synaptic devices using semiconductor CNTs and CsPbBr_3_-QDs. The transistor demonstrated light intensity-dependent synaptic plasticity, enabling it to emulate the learning and memory functions of the human brain. It is noteworthy that the authors fabricated a 32 × 32 pixel synaptic array, as shown in Fig. 9a–c for the integration of array chips onto a printed circuit board, including bonding lines and interconnects within the circuitry, as well as individual synaptic devices. The array further showcased an exceptional device yield of 100%, underscoring its superior image sensing and memory capabilities. Consequently, leveraging the synaptic plasticity of the devices within the array, they successfully simulated the distinctiveness of familiar human faces compared to unfamiliar ones, as illustrated in Fig. 9d. The synaptic array was trained using varying numbers of pulses of ultra-weak light (1 μW cm^−2^), resulting in weight plots that exhibited increasing similarity and enhanced image sharpness (Fig. 9e). Additionally, as shown in Fig. 9f, an increase in light intensity could expedite the process of pattern acquisition. This scenario was analogous to interpersonal communication, where increased interaction with an individual led to a stronger impression of their facial features in memory. The evolution of the face learning process was simulated based on the experimental synaptic properties depicted in Fig. 9e, f. As illustrated in Fig. 9g, an increase in the number of training pulses resulted in a greater acquisition of facial features [143].

**Fig. 10:**
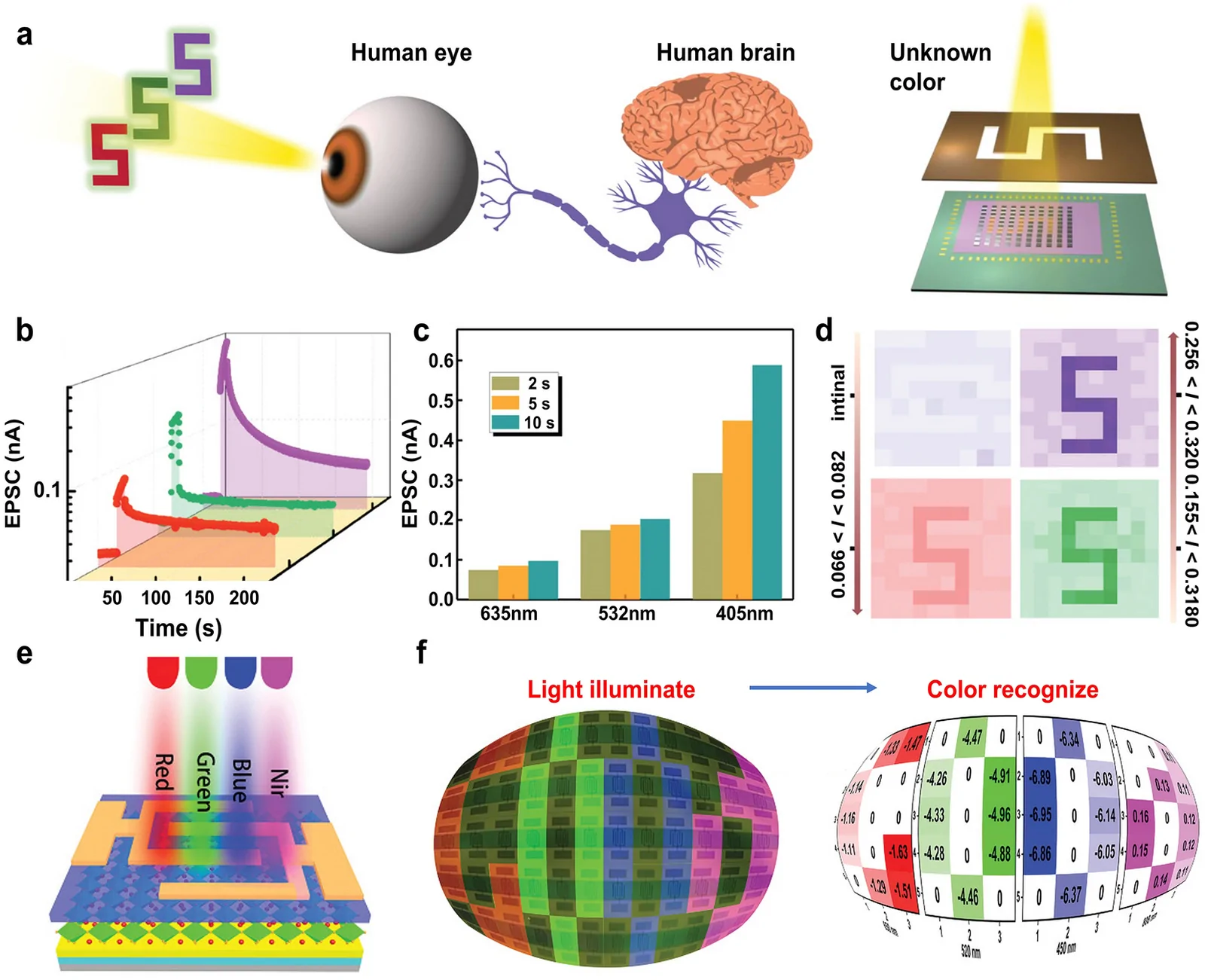
**a** Schematic representation of the color recognition process by which the human visual system and synaptic devices. **b, c** EPSC values triggered by multi-color light for different durations. **d** Diagram of the recognition results. Reproduced from Ref. [122] with permission from John Wiley and Sons, Copyright 2022. **e** Schematic of a transistor synapse illuminated by four colors of light. **f** Results of color recognition. Reproduced from Ref. [130] with permission from John Wiley and Sons, Copyright 2021

Furthermore, leveraging their differential sensitivity to incident light of different wavelengths, perovskite synapses can generate postsynaptic currents with varying intensities, thus achieving color recognition function. This capability could improve the efficiency of subsequent processing tasks and boosting the accuracy of image recognition [177]. Utilizing lead-free perovskite Cs_3_Bi_2_I_9_ as photosensitive material, a 10 × 10 pixel floating-gate synaptic device array was fabricated to facilitate the image preprocessing stage for color recognition [122]. The process by which the human visual system and synaptic apparatus recognize unfamiliar light stimuli is illustrated in Fig. 10a. The light passed through a mask containing the letter “5” to obtain a patterned light with the number “5”. As displayed in Fig. 10b, c, owing to the differential photosensitivity of the absorption layer to different light wavelengths, the observed postsynaptic currents were 588, 202, and 98 pA, respectively, upon irradiating the devices with 405, 532, and 635 nm light at a consistent power intensity (0.1 mW cm^−2^) and duration (10 s). Furthermore, the EPSC and the differences between them demonstrated a notable increase with increasing light duration (2, 5, and 10 s). As shown in Fig. 10d, a distinguishable pattern was exhibited, and this feature could be used to identify numbers or letters with different colors, demonstrating its ability to mimic the function of the human eye’s retina by distinguishing colors. Using the gate as an additional modulation terminal, preprocessing functions for discriminating multiple colors were achieved via a transistorized synaptic array fabricated based on PEA_2_SnI_4_ and Y6, which was employed to exhibit this functionality (Fig. 10e) [130]. The researchers observed that when V_DS_ and V_GS_ were set to − 40 V, light stimulation with red (650 nm), green (520 nm), and blue (450 nm) light stimuli resulted in larger currents, which is characteristic of EPSC. In contrast, it was unexpected that when V_DS_ and V_GS_ were positive (40 V), irradiation with red, green, and blue light resulted in smaller currents, indicative of inhibitory postsynaptic current (IPSC) behavior. Conversely, stimulation with near-infrared (NIR) light (808 nm) yielded EPSC. Distinct postsynaptic currents can be generated by sequentially illuminating different colors of light with an identical intensity of 25 µW cm^−2^ on the device at a V_DS_ of 40 V. Accordingly, for an unidentified type of incident light, the internal processor was capable of distinguishing the color of the light based on the type of postsynaptic current and the magnitude of the ΔPSC value. Four light-emitting diodes (LEDs) of distinct colors were employed to illuminate the synaptic array by transmitting light through the four letters “G”, “O”, “O” and “D”. The final calculated current intensity was displayed on each pixel, indicating that the synaptic transistor array was capable of not only discerning the color of light, but also displaying an image of four different colored characters (Fig. 10f). The findings illustrate that optoelectronic synaptic devices are capable of replicating the color-recognition capabilities of the human retina.

In sum, the high sensitivity and multi-color response characteristics of perovskite synapses present advantages for imaging technologies based on biomimetic synaptic devices. Perovskite synapses enable preprocessing functions at the sensor level, including image sensing, contrast enhancement, and color recognition. This capability establishes the foundation for subsequent image processing tasks (e.g., image recognition and motion detection) within neuromorphic visual computing systems.

### Visual Adaptation

**Fig. 11:**
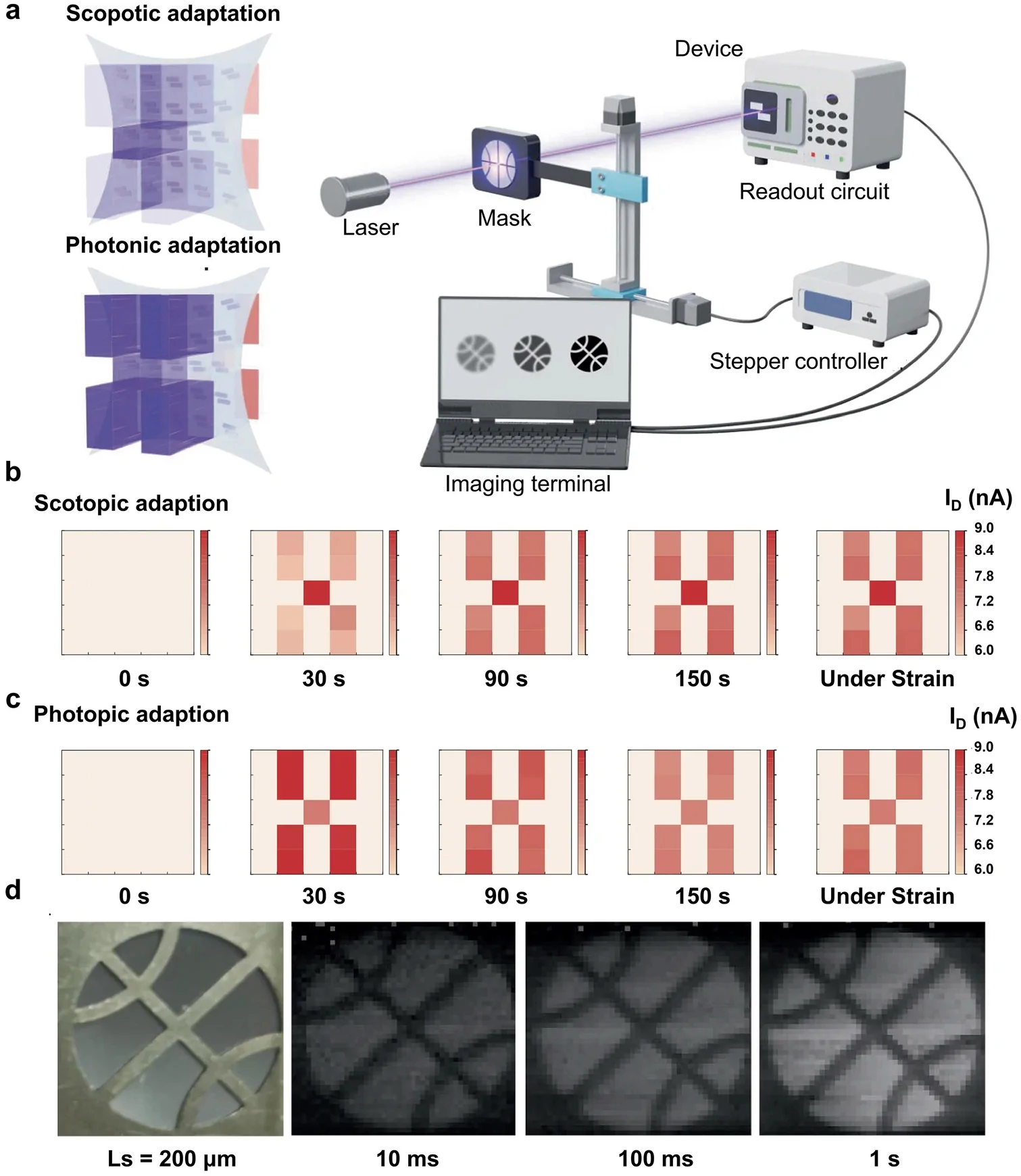
**a** Schematic of synaptic device array and imaging. **b, c** Dark-adapted and bright-adapted behaviors of the array under different conditions. **d** Simulated visual adaptation results. Reproduced from Ref. [111] with permission from Springer Nature, Copyright 2024

Perovskite synapses can further integrate the photoreceptive functions of rod cells and cone cells in the human visual system, enabling dynamic adaptation to varying light intensities. This significantly enhances the processing efficiency, reliability, and compactness of visual computing systems, providing transformative opportunities for developing next-generation neuromorphic imaging systems with intelligent processing capabilities [178]. This has been demonstrated in a stretchable transistorized synaptic device that used viscoelastic photosensitive membranes fabricated from CsPbBr_3_ quantum dots and SEBS elastomers [111]. During the preparation of hybrid perovskite membranes, of particular importance is the introduction and adaptation of defective states, which plays a pivotal role in subsequent adaptation processes. The drain current displayed notable tunability in its growth and decay processes, exhibiting alterations in response to diverse light conditions with varying light intensities (6.41, 356.69, and 698.09 μW cm^−2^, corresponding to dim to bright) and gate bias (−10, 0, and + 10 V). Subsequently, the adaptation behavior of the devices was quantified using the current change ratio (CCR) index, which was defined as the ratio of the source-drain current at 50 s of illumination and to that at 10 s. CCR value greater than 1 typically signified photocurrent excitation, whereas value less than 1 indicated photocurrent suppression. The highest CCR value of 1.68 for dark adaptation was attained in dim light at 6.41 μW cm^−2^, while the lowest CCR value of 0.74 for bright vision adaptation was observed at 698.09 μW cm^−2^, V_G_ of 0 V. The CCR index indicated a correlation between the gate voltage and the transition from a state with a value less than 1 to one with a value greater than 1 as the voltage was adjusted from − 10 to 10 V. Thus, by adjusting the light intensity and gate voltage, the device could achieve different adaptive behaviors. A 5 × 5 pixel transistor array and a distinctive “X” configuration were employed to emulate the visual adaptation functionality of the human eye (Fig. 11a). The center pixel of the pattern was illuminated by an appropriate background light (normal: 356.69 μW cm^−2^) for reference and compared with the outer eight pixels (bright: 698.09 μW cm^−2^ or dim: 6.41 μW cm^−2^). As shown in Fig. 11b, c, the contrast of the X-shaped image was enhanced over time under dim light conditions. In contrast, the corresponding distributions became blurred when the illumination was bright. The array exhibited a markedly higher response time (< 150 s), which was considerably faster than the time required by the human eye (3–30 min). The synaptic array maintained visual adaptation functionality even at 50% stretching strain. This function displayed a dependence on gate voltage, with higher image contrast at positive gate voltage. Further, a single-pixel test system featuring a stepping platform and readout circuit was employed to demonstrate the adaptive imaging of complex objects. As illustrated in Fig. 11d, the images of the basketballs display heightened contrast and enhanced clarity over time. The effective visual adaptability and stretchability exhibited by these optoelectronic synaptic devices are anticipated to be designated an intelligent neuromorphic electronic device, thereby paving the way for advanced visualization applications, dermatoid artificial intelligence devices, and bionic robots.

### Pattern Recognition

**Fig. 12:**
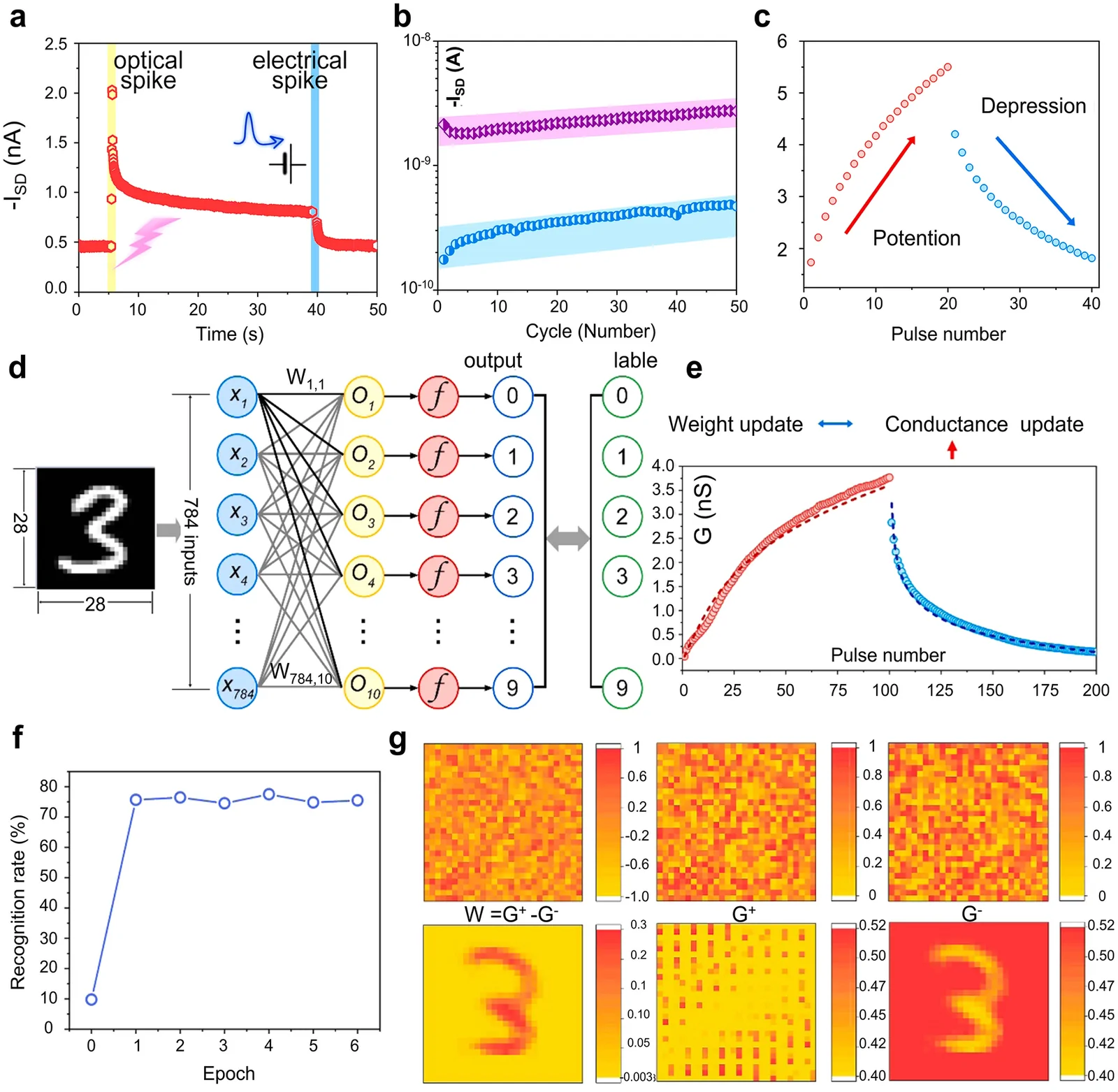
**a** Optical enhancement and electrical inhibition processes. **b** Reproducible performance of the device. **c** LTP and LTD behavior resulting from 20 optical/electrical stimuli. **d** Schematic representation of the recognition process of ANN. **e** LTP and LTD behavior resulting from 100 optical/electrical stimuli. **f** Recognition accuracy varies as a result of the training process. **g** Mapping of conductance and synaptic weights. Reproduced from Ref. [61] with permission from Elsevier, Copyright 2021

Leveraging the flexibility of photoelectric modulation, perovskite synapses not only eliminate redundant sensing devices but also exhibit LTP and LTD behaviors with good linearity, enabling them to perform pattern recognition tasks. A synaptic transistor consisting of the organic semiconductor material C8-BTBT, the polymer PS, and the CsPbBr_3_ quantum dots has been developed to process visual information [61]. The devices demonstrated the capacity to observe EPSC behavior under extreme conditions of optical spike stimulation (365 nm), with a duration of 0.01 s, an intensity of 0.5 μW cm^−2^, and a readout voltage of − 0.01 V, the calculated energy consumption was 0.11 fJ. The synaptic devices also showed a response to optical inputs at wavelengths of 450 and 500 nm. The transition from STP to LTP behavior could be achieved by increasing the intensity and duration of the light stimulus. The application of an electrical pulse to the gate electrode could result in a reduction in the current, which could be regarded as an IPSC. As illustrated in Fig. 12a, the current exhibited an initial increase followed by a gradual decline following the application of a light pulse (duration of 0.1 s, intensity of 10 μW cm^−2^). This response can be reversed by the subsequent application of a negative − 10 V electrical spike for 0.3 s, thereby recovering the original current state. The reproducible performance of the device was demonstrated by a configuration of EPSC and IPSC formed by the alternating application of 50 optical and electrical pulses, as shown in Fig. 12b. The step increased and decreased characteristics of the current under 20 optical stimuli (intensity of 10 μW cm^−2^, duration of 0.1 s, interval of 0.9 s) and 20 electrical stimuli (intensity of − 10 V, duration of 0.06 s, interval of 18 s) are shown in Fig. 12c. The favorable characteristics of this combination could be attributed to the high sensitivity of perovskite quantum dots and PS polymers as well as their ability to facilitate efficient separation of photogenerated charges. The conventional multilayer perceptron neural network, as illustrated in Fig. 12d, was subsequently constructed and employed for the recognition process. Utilizing the curve of weight updates for 100 light/electrical pulse stimuli depicted in Fig. 12e, the recognition accuracy remained consistent at approximately 75% after 6 training epochs as depicted in Fig. 12f. The synaptic weight mapping and corresponding conductance before and after training are visually represented in Fig. 12g.

**Fig. 13:**
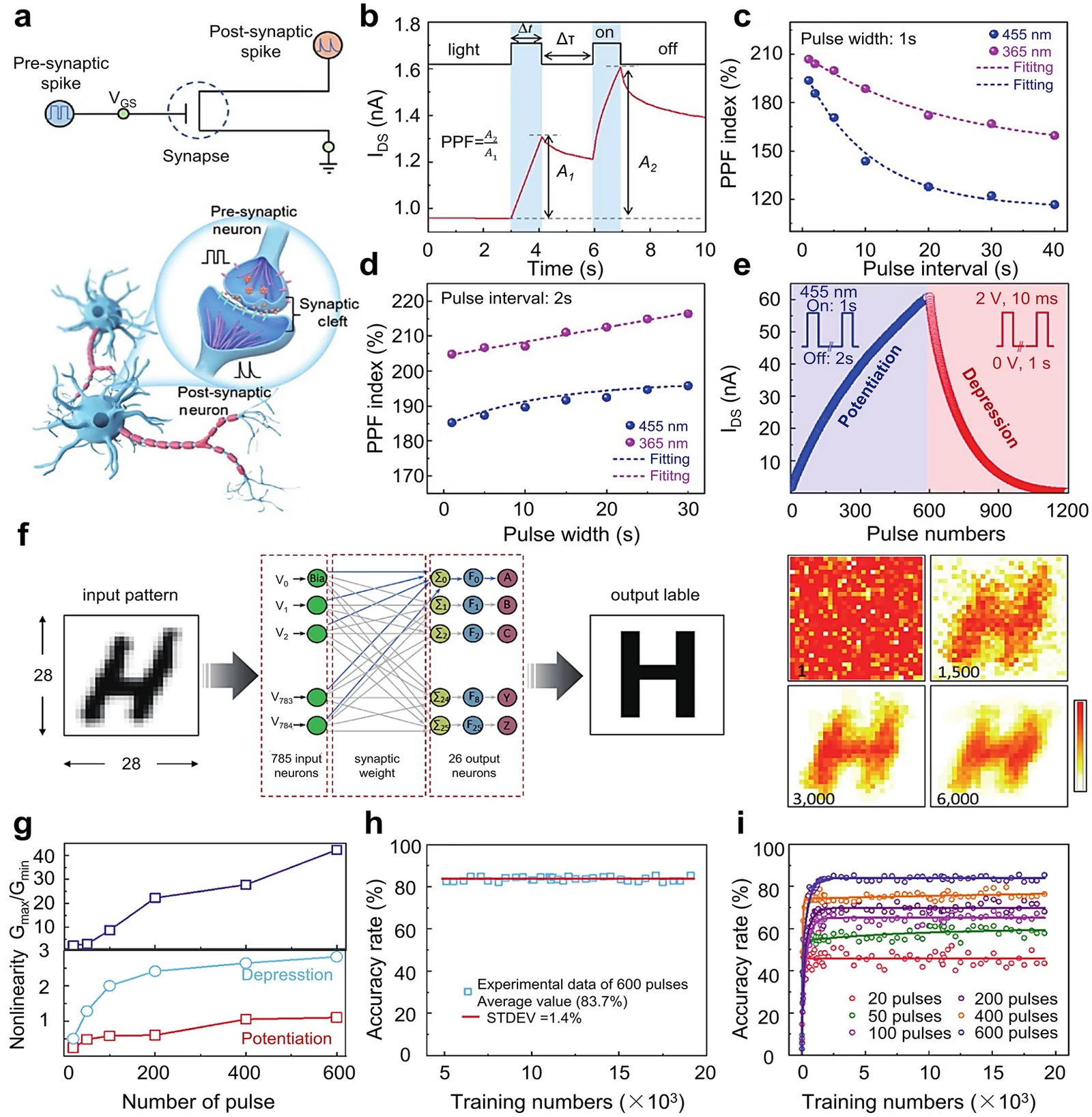
**a** Schematic representation of artificial synaptic devices and biological synapses. **b** PPF behavior. **c, d** PPF index as a function of light duration and pulse interval under different wavelengths of incident light stimulation. **e** LTP and LTD behavior obtained with 600 enhancement/suppression pulses. **f** Schematic representation of letter recognition process. **g** Calculated nonlinearity and dynamic range as a function of the number of pulses. **h** Highest recognition accuracy of artificial neural networks. **i** Recognition accuracies obtained with different numbers of pulses. Reproduced from Ref. [127] with permission from Springer Nature, Copyright 2022

By tuning the programming input to balance the dynamic range and linearity, the accuracy of perovskite synapses in digit recognition can be further improved. This observation was corroborated in a study that constructed 3 T synaptic devices using single-crystal CsAgBiBr_6_ and IGZO [127]. The application of voltage and light stimuli as presynaptic stimuli enabled synaptic devices to exhibit a diverse range of synaptic behaviors (Fig. 13a). As illustrated in Fig. 13b, the device was capable of exhibiting PPF behavior when two paired optical pulses were applied. It could be found that the PPF index decreased with the increase of the pulse interval time and increased with increasing pulse width. The PPF index was at its maximum under irradiation with light of a wavelength of 365 nm, with a potential to exceed 210% (Fig. 13c, d). The transition from STP to LTP was achieved through a series of modifications, encompassing the utilization of heightened light intensity, expanded pulse widths, and variations in the number of light pulses. The weight update curves for LTP and LTD were obtained by employing optical enhancement pulses at a wavelength of 455 nm, an intensity of 12.7 mW cm^−2^, a duration of 1 s with an interval of 2 s, as well as electrical suppression pulses with an intensity of 2 V, a duration of 10 ms, and an interval of 1 s (Fig. 13e). The researchers concluded that the favorable visible light absorption of single-crystal perovskite and the efficient charge transfer at the heterojunction interface enhanced the photo response and synaptic plasticity of the devices. Subsequently, the accuracy of the classification pattern was evaluated through the utilization of the single-layer perceptron simulator. The contrast and shape of the output number “H” become more pronounced and discernible as the training epoch increased from 1 to 6,000 (Fig. 13f). And as illustrated in Fig. 13g, the dynamic range and nonlinearity demonstrate a gradual increased with the number of pulses applied to the stimulus. The recognition accuracy achieved on the basis of LTP and LTD realized with different number of pulses is shown in Fig. 13h, i. It was noteworthy that although the nonlinearity of enhancement and suppression exhibited the lowest value at 20 optical pulses, the dynamic range was insufficient to achieve high accuracy. The highest level of recognition accuracy, approximately 83.8%, was achieved with 600 pulses when the dynamic range was significantly expanded.

To attain higher recognition accuracy, not only is the optimization of synaptic plasticity necessary, but the improvement of algorithms should also not be overlooked. By using lead-free perovskite CsBi_3_I_10_ as the photosensitive floating-gate material, PDPP4T as the semiconductor channel material, and PVP as the tunneling layer material, a floating-gate-type synaptic device was fabricated [62]. It was posited that the training process of the neural network was hindered when synaptic weights were represented by the difference in conductance between two synaptic devices due to the limited conductance state of a single device. They developed a method employing six synaptic devices to represent multi-digit weights. Following 40 training periods, the recognition rate eventually reached 91.85%.

**Fig. 14:**
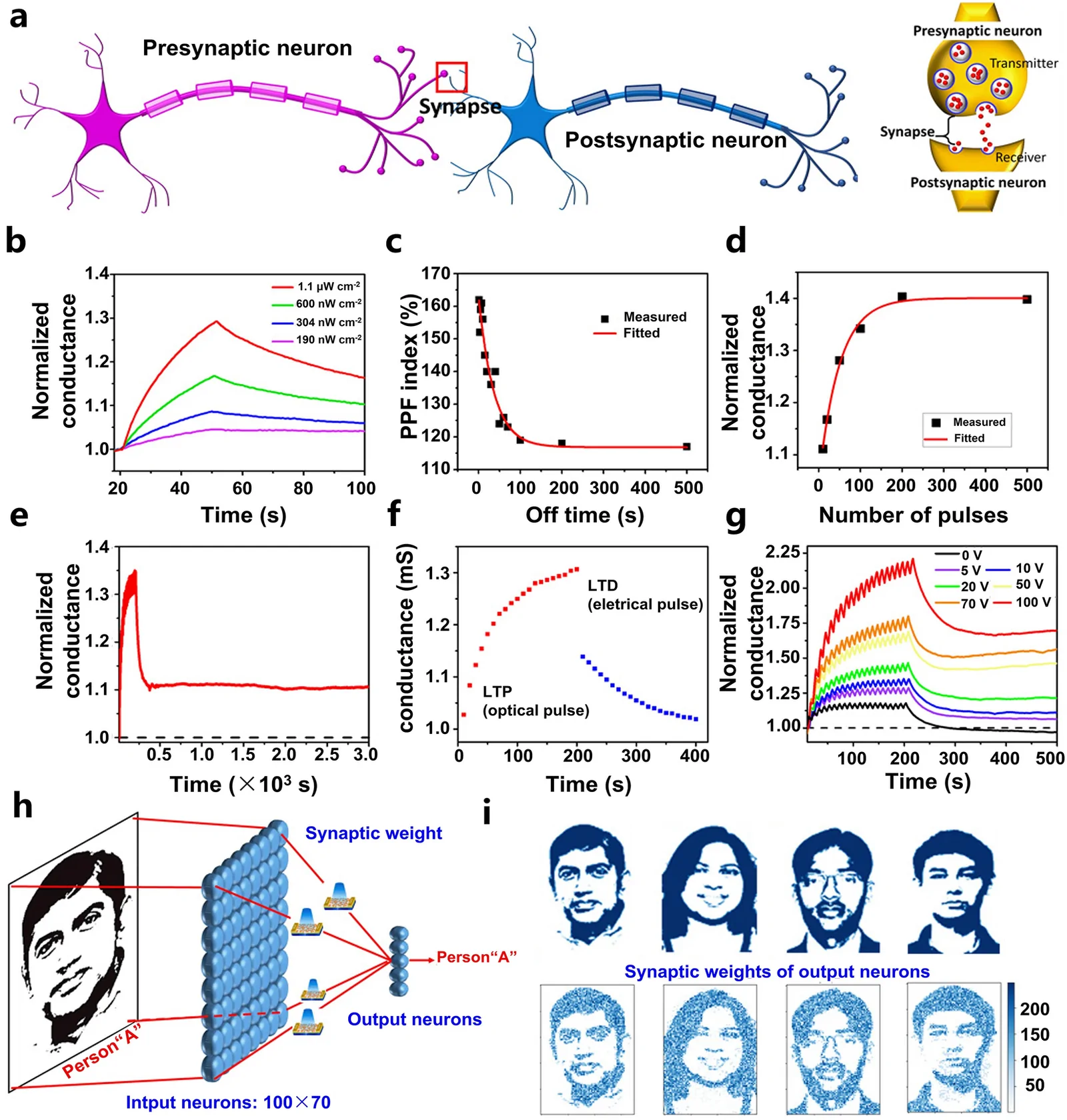
**a** Schematic of human neurons and biological synapses. **b** Variation of device conductance due to a single pulse. **c** PPF behavior as a function of pulse interval. **d** Changes in device conductance with the number of pulses. **e** Variation of the conductance due to 20 optical pulses.** f** LTP and LTD characteristic due to the optical pulses and drain voltage pulses. **g** Gate-dependent conductance characteristics of the device. **h** Face recognition based on neural networks. **i** Synaptic weights of real images and corresponding output neurons. Reproduced from Ref. [137] with permission from American Association for the Advancement of Science, Copyright 2021

Similarly, by leveraging the long-term plasticity of perovskite synapses, face recognition—an important research direction in the field of pattern recognition—can also be implemented in perovskite synapses. Using MAPbBr_3_ quantum dots grown from a graphene lattice as the photosensitive layer, a transistor-type synaptic device was fabricated [137]. The authors posited that the grown perovskite quantum dots facilitate more efficient charge transfer than the heterogeneous structure obtained by spin coating. Consequently, the device demonstrated a responsivity of 1.4 × 10^8^ A W^−1^ and a specific detectivity of 4.72 × 10^15^ Jones at an optical wavelength of 430 nm. Subsequently, the synaptic behavior of the device was examined, wherein the light pulse served as a presynaptic signal, and the current obtained in the channel was regarded as a postsynaptic signal (Fig. 14a). The highest intensity (1.1 μW cm^−2^, duration of 30 s) of light at V_D__S_ of 0.5 V and V_G_ of 10 V, applied as a single pulse, resulted in a greater level of conductance (Fig. 14b). For the application of paired pulses (duration of 5 s), the PPF index also exhibits a decrease with an increase in the pulse interval (Fig. 14c). As shown in Fig. 14d, the device’s conductance demonstrated an increase with the number of pulses (5 s for both duration and interval), reaching a saturation point at 20 pulses. The alteration in normalized conductance in response to 20 presynaptic light spikes is illustrated in Fig. 14e. Upon cessation of illumination, the conductance did not recover to its initial state, and this state persisted for up to 3000 s. In contrast to conventional devices, the LTP and LTD behavior of the device was obtained by applying a series of optical pulses (5 s for both duration and interval) and a series of drain electrical pulses (− 0.5 V, 1 s for both duration and interval) at V_G_ = 10 V (Fig. 14f). Also, it was demonstrated that the LTP behavior was influenced by the gate bias voltage, which can be attributed to the fact that the electron capture-induced quasi-p-doping of graphene was more pronounced when the gate voltage was higher (Fig. 14g). Subsequently, as presented in Fig. 14h, i, a spiking neural network was constructed to perform the face recognition task based on the fitted conductance properties of the device. The trained synaptic weight images showed a high correlation with the features present in the real portrait, thereby substantiating the recognition capability of synaptic devices within this unsupervised spiking neural network.

**Fig. 15:**
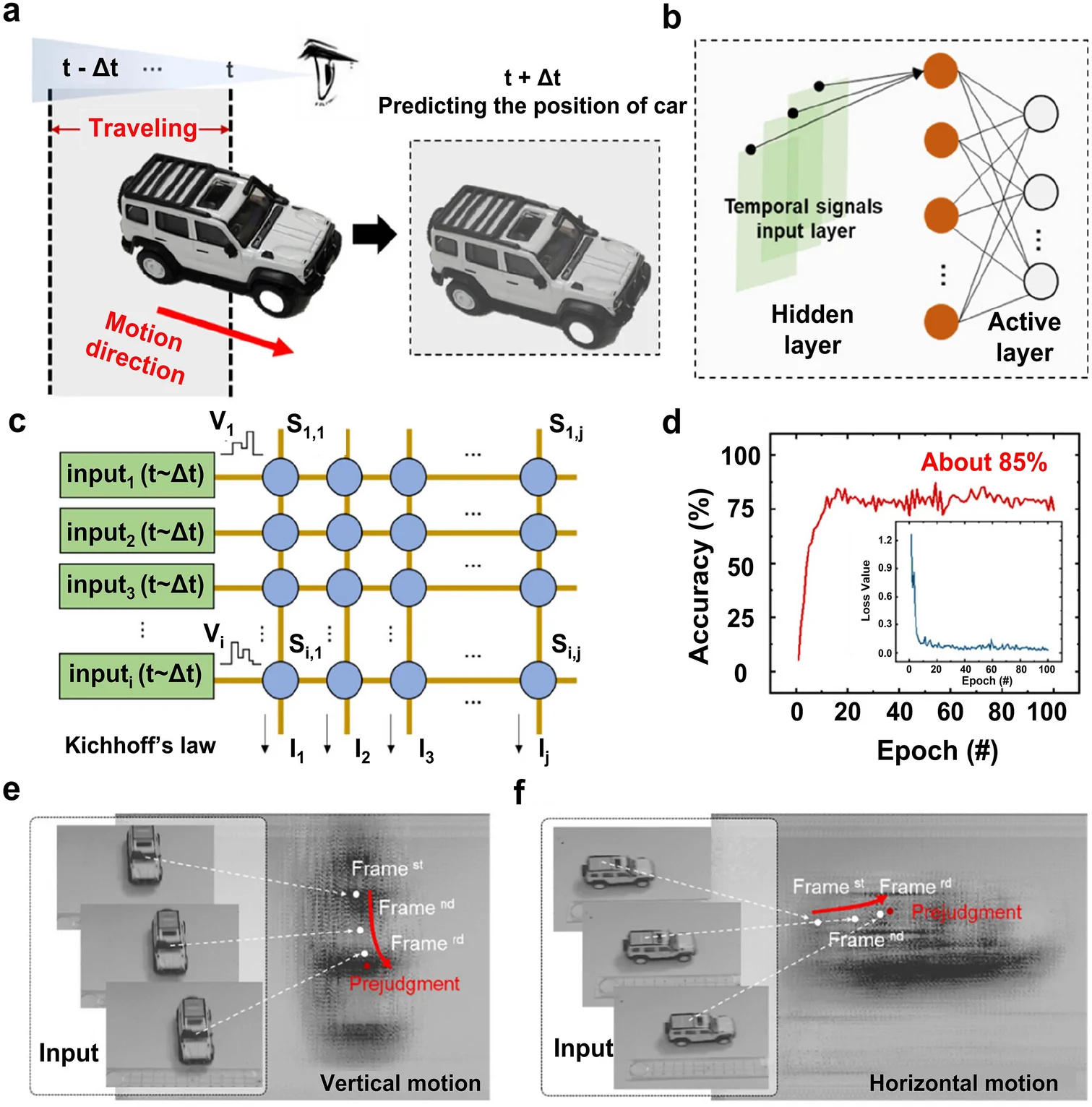
**a, b** Composition of the hardware system and schematic diagram of predicting the trajectory of a moving object. **c** Hidden layer of the device-based array circuit. **d** Training accuracy of hardware systems. **e, f** Results of predicting vertically moving cars and horizontally moving cars. Reproduced from Ref. [131] with permission from American Chemical Society, Copyright 2024

Utilizing their time-dependent synaptic plasticity, perovskite synapses can be further utilized to process time-related motion information, thereby achieving motion recognition function. A hardware system for motion recognition has been developed [131]. Perovskite synapses were constructed by using CsPbBrI_2_ nanowires and PDVT-10 heterojunctions, and the dynamic response characteristics of the devices were analyzed. The researchers observed that synaptic behaviors, such as PPF, could be elicited using gate electrical pulses. They noted a tendency for the postsynaptic currents to increase with an increase in the width, intensity, and number of electrical pulses. Compared to the condition without illumination, the postsynaptic current intensity increased by 30 times when the device was exposed to UV light (365 nm). In the field of biology, it has been observed that when a large voltage stimulus is applied at different times, the synaptic weights are found to be greater when the period of application is closer to the post pulse period. The above dynamic response behavior to time-varying signals was also verified in the synaptic device. Specifically, four electrical pulses of different amplitudes (18, 20, 22, and 24 V) were treated as smaller stimuli, while the light pulse simulation was regarded as the larger stimulus. In the case of applying four electrical pulses and one optical pulse, the later the application time of the optical pulse, the higher the current. Furthermore, when two light pulses were applied, it was found that the current obtained by applying light pulses to the first and third electrical pulses approximated the current obtained by applying a single light pulse to the fourth electrical pulse. This implied that the device’s response could be modulated by manipulating both the amplitude and temporal characteristics of the input stimulus. In light of this dynamic response property, a hardware system based on perovskite synapses was utilized to forecast the trajectory of a moving object, comprising primarily a temporal signal input layer, a hidden layer, and an activity layer (Fig. 15a, b). Initially, the trained weights were mapped to the array circuit by writing lines. Subsequently, the input video was encoded as a sequence of optical pulses, which were then applied to the trained array circuit (Fig. 15c). This indicated that visual input data was partitioned into a sequence of time frames. The calculation of the final frame was based on the output of the preceding frame. The calculated feature frames were stored until the last frame was reached, at which point they were fused to yield a prediction. Ultimately, the output currents in various branches of the array circuit would accumulate in accordance with Kirchhoff’s law. The final training accuracy obtained was about 85% (Fig. 15d). The results of trajectory prediction for a moving car are shown in Fig. 15e, f, demonstrating the successful anticipation of the car’s path under uniform deceleration in both vertical and horizontal directions.

**Fig. 16:**
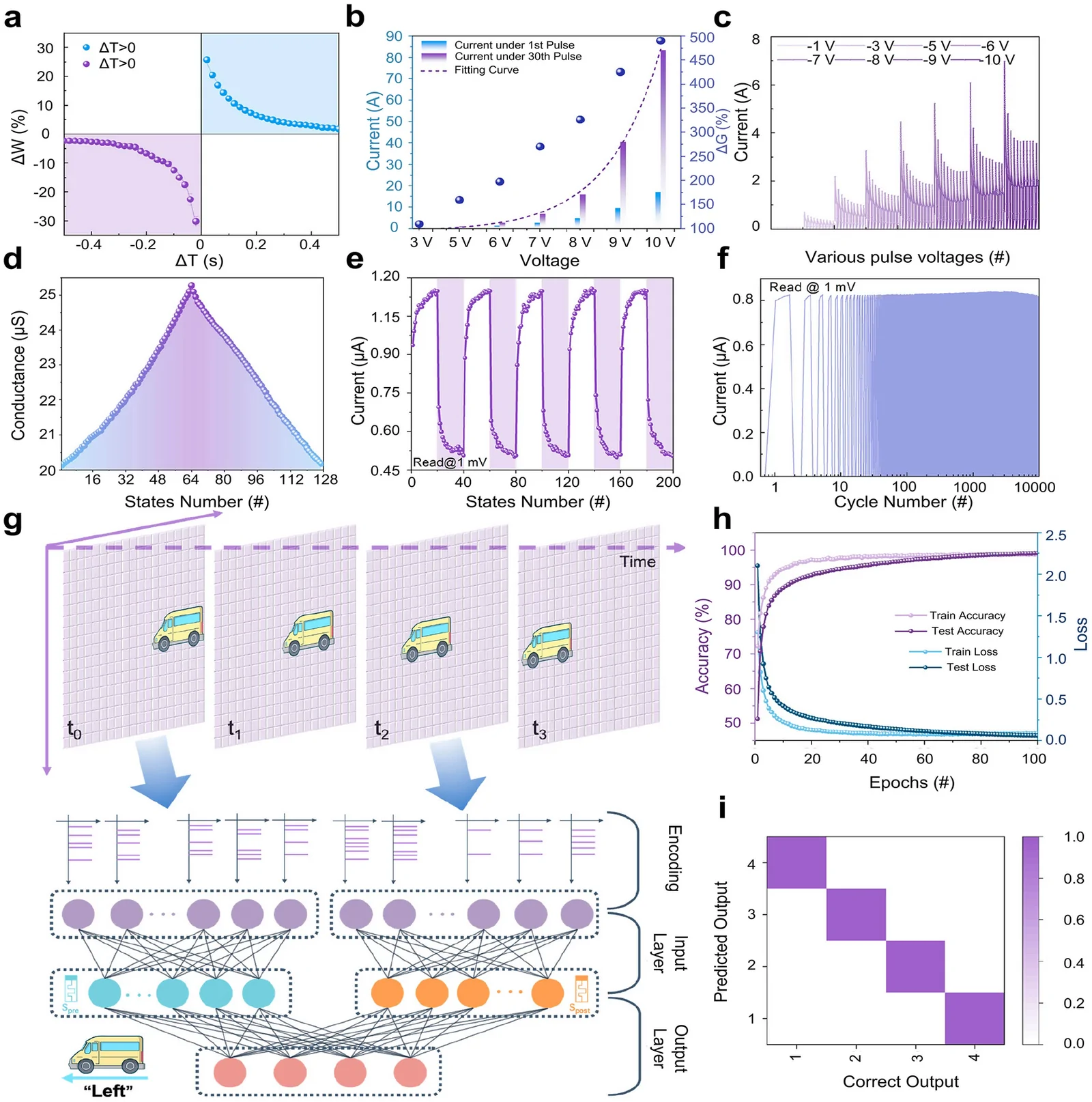
**a** The STDP characteristics of the device. **b, c** Variation of device response current with voltage change under positive/negative electrical modulation. **d** LTP and LTD characteristics of the device. **e** Device current for 5 enhancement/suppression cycles.** f** Stability of the device under light irradiation. **g** Schematic of the SNN network used to recognize the direction of car motion. **h** Recognition accuracy after training. **i** Corresponding confusion matrix of the recognition. Reproduced from Ref. [179] with permission from American Chemical Society, Copyright 2024

In addition, owing to the successful simulation of STDP behavior, perovskite synapses can also function in spiking neural networks, enabling directional motion direction recognition in a different manner. This has been demonstrated in the 2 T devices, where the Au and the ITO corresponded to the presynaptic neuron and postsynaptic neuron, respectively [179]. The halide perovskite MA_0.825_FA_0.275_Pb_0.75_Sn_0.25_I_3_ semiconductor layer, situated in the middle of the device, corresponded to the synaptic gaps and provided a platform for realizing synaptic behaviors and visual information processing. Furthermore, BCP/C60 and PEDOT:PSS were introduced at the interface between the perovskite layer and the top and bottom electrodes, which could enhance the optical response while maintaining the synaptic properties. It was noteworthy that the various synaptic behaviors were achieved through the modulation of a photoelectrically coupled stimulus. The generation of PPF behavior could be achieved through the application of electrical pulses with a pulse width of 2 s and an amplitude of 0.5 V (V_read_ of 0.1 V) under 405 nm light irradiation with an optical power of 2.5 mW mm^−2^. The STDP behavior, which characterizes the impact of the relative temporal relationship between presynaptic and postsynaptic action potentials on synaptic connection strength, was simulated as depicted in Fig. 16a. Specifically, a fixed pulse amplitude of 0.5 V and width of 200 ms were employed while varying the time interval (Δt) between pre- and postsynaptic spike. It was demonstrated that LTD behavior was observed when Δt < 0, whereas LTP occurred when Δt > 0, in accordance with the Hebbian rule. As can be seen in Fig. 16b, c, the current also demonstrated an exponential increase with pulse amplitude (from 3 to 10 V) and can be erased by the application of a negative voltage pulse (pulse width of 15 μs, and interval of 3 μs.). The application of write and erase electrical spikes (± 0.5 V, 100 ms) when the device was under light conditions enabled the attainment of long-term synaptic plasticity with favorable linearity and symmetry (Fig. 16d). Furthermore, the device demonstrated consistent and reliable performance even after the application of 20 voltage pulses for 5 consecutive cycles for potentiation and inhibition, as well as over 10^4^ pulses (Fig. 16e, f). The STDP behavior of the optoelectronic synaptic device aligns with the information transmission pattern of the spiking neural network (SNN), enabling the SNN to encode temporal information and, consequently, to be utilized for motion recognition. A SNN was constructed based on the STDP properties of the synaptic device, as illustrated in Fig. 16g. When moving objects passed by, paired pulse signals were generated, which were subsequently fed to the presynaptic and postsynaptic neurons, respectively. The synaptic weights were subject to variation in accordance with the pulse time interval, enabling the SNN to learn relevant features in real time. Ultimately, the direction of motion of the vehicle could be represented by the corresponding labels of the neurons. The recognition accuracy for the rightward motion pavilion and the overall four directions are presented in Fig. 16h, i. The implemented recognition method, utilizing SNN based on optoelectronic synaptic devices, not only achieved a perception accuracy of 99.1% in motion direction but also concurrently enhanced computational speed and reduced energy consumption.

In conclusion, perovskite synapses are capable of not only detecting optical information but also processing signals in real time and storing the results, which enables the construction of neuromorphic vision systems with sensing-memory-computing capabilities. Neuromorphic vision systems based on perovskite synapses have progressively acquired complex functionalities analogous to human vision through continuous development and advancement. Building upon the foundational functions of imaging and visual information preprocessing, they have now evolved to encompass critical capabilities such as visual adaptation and pattern recognition. Nevertheless, the current research still faces numerous challenges before practical application. On one hand, in addition to the pursuit of higher sensitivity and more functional integration, enhancing the efficiency and accuracy of visual information processing remains a key direction for future development. Furthermore, hardware implementation also demands immediate consideration, which will be the focus of the next chapter.

## Toward Perovskite Synapses-Based Neuromorphic Systems

Significant progress has been made in enhancing the performance of biomimetic synaptic devices through materials and device engineering. Meanwhile, several studies have presented energy-efficient and high-performance data processing solutions via neuromorphic computing systems based on perovskite synapses. However, realizing practical neuromorphic systems suitable for real-world applications remains challenging. Bridging this gap requires addressing several challenges associated with synaptic devices, ranging from material preparation, device design to hardware implementation. This chapter focuses on the implementation of perovskite-based neuromorphic vision computing systems at the array and hardware integration level, summarizes the latest research developments, examines the potential challenges and outline potential research directions.

Current research progress in perovskite synapses-based neuromorphic vision computing systems is mainly limited to the construction of perovskite synapses arrays, with a few studies exploring the integration of small-scale arrays with other units.

**Fig. 17:**
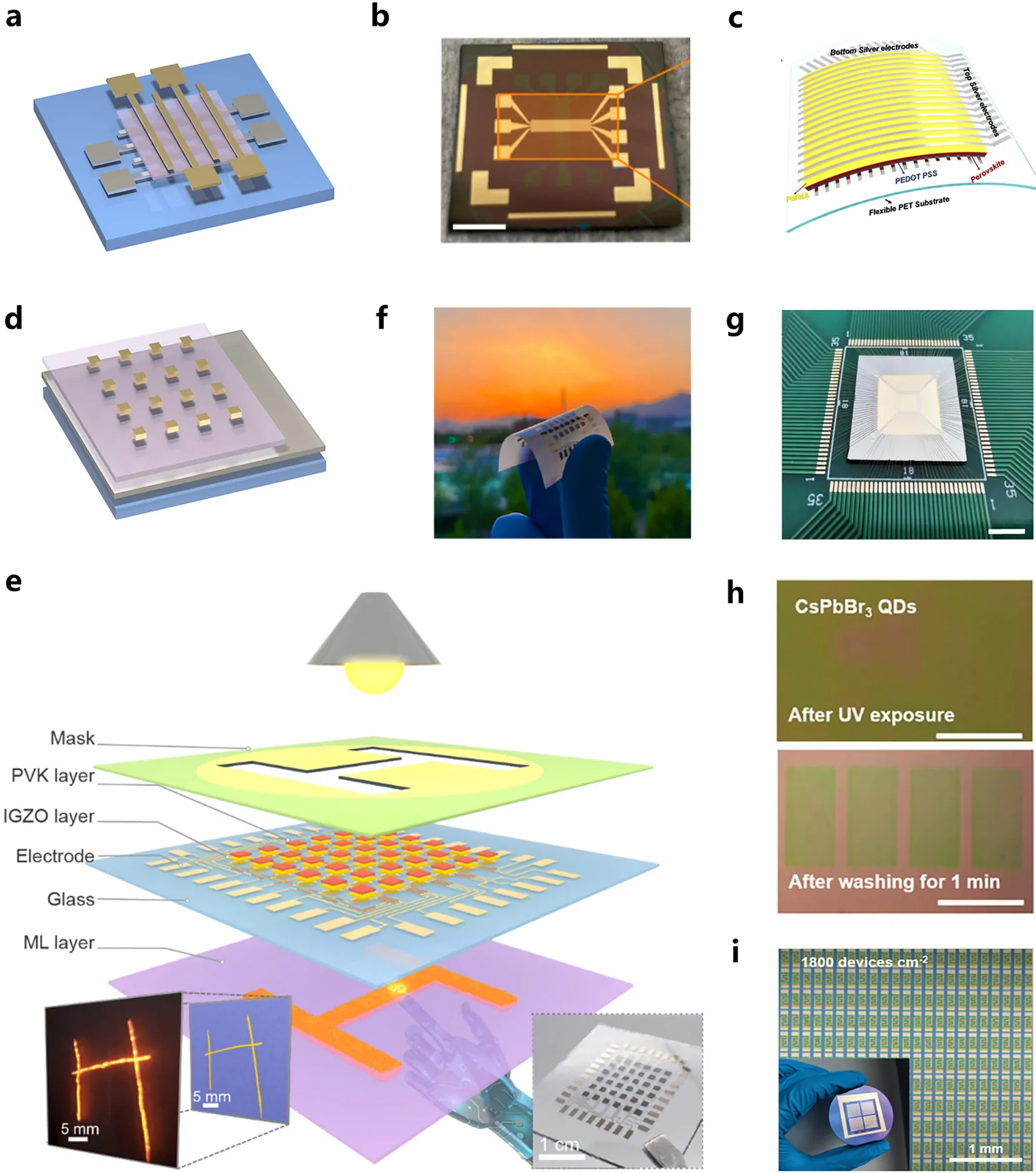
**a** Schematic diagram of crossbar array. Reproduced from Ref. [181] with permission from John Wiley and Sons, Copyright 2024. **b** 7 × 7-pixel crossbar array. Reproduced from Ref. [73] with permission from Springer Nature, Copyright 2024. **c** 16 × 16-pixel array on a flexible substrate. Reproduced from Ref. [71] with permission from Royal Society of Chemistry, Copyright 2024. **d** Schematic diagram of dot array. Reproduced from Ref. [181] with permission from John Wiley and Sons, Copyright 2024. **e** Schematic of the synapse array device and the photograph of the array. **f** Flexible synapse array. Reproduced from Ref. [182] with permission from John Wiley and Sons, Copyright 2023. **g** 32 × 32-pixel dot array. Reproduced from Ref. [143] with permission from Springer Nature, Copyright 2021. **h** Optical images of perovskite films after UV exposure and washing for 1 min. **i** Optical image of high-density perovskite synapse array. Reproduced from Ref. [148] with permission from John Wiley and Sons, Copyright 2024

Regarding array fabrication, 2 T perovskite synapses have advanced swiftly from initial dot arrays to crossbar arrays. Dot arrays, featuring discrete devices with shared bottom electrodes that typically patterned through shadow mask, offer fabrication simplicity. Therefore, synaptic device arrays based on this structure have been widely reported in perovskite synapses [75, 95]. As another configuration for synaptic arrays, the crossbar array comprising multiple rows of top and bottom electrodes that form an interconnected network of intersecting points, as shown in Fig. 17a, are being more widely documented in perovskite synapses. In this configuration, artificial neurons were represented by word and bit lines, while the center denoted biomimetic synapses. It offers reconfigurable non-volatile resistance states and could reducing power dissipation in vector–matrix multiplication, a core computing task in signal and image processing [180]. Owing to its ability to offer the most precise characterization of device performance upon integration, it gains significant favor in commercial research [181]. Such architecture in most instances is manufactured via shadow masks with a 90° offset, or in some cases, though patterned ITO as the bottom electrodes. As depicted in Fig. 17b, a 7 × 7-pixel crossbar array with a yield of 100% that demonstrate idea analog programming capabilities has been fabricated by e-beam evaporation using a shadow mask [73]. This architecture can also be implemented on a flexible substrate, as shown in Fig. 17c [71].

For 3 T perovskite synapses, dot arrays remain the primary choice with the array scale tending to increase (Fig. 17d). A 6 × 6-unit perovskite synapse array fabricated via shadow mask has also been reported with a thin polydimethylsiloxane (PDMS) layer adopted for packaging to boost device stability, as shown in Fig. 17e [182]. The array can also be fabricated on a flexible substrate, facilitating its integration into mechanically compliant systems (Fig. 17f). Moreover, based on a simple bottom gate bottom contact device structure that does not require additional patterning of perovskite materials, researchers have fabricated a 32 × 32 pixel synaptic array, as shown in Fig. 17g for the integration of array chips onto a printed circuit board [143]. Furthermore, to develop synaptic arrays with higher density, an effective cross-linking microlithography strategy has been reported for fabricating patterned perovskite synapse arrays [148]. As shown in Fig. 17h, exposing the functional film mixed with a photo cross-linkable-azide-crosslinker to UV light via a photomask allows for the selective enhancement of the film’s solvent resistance, thereby enabling patterning through solvent washing. Perovskite synapse array with a density of 1800 devices cm^−2^ was successfully fabricated, shown in Fig. 17i. The array also exhibits excellent uniformity and yield, with 100 randomly chosen devices functioning properly, which suggests an almost 100% device yield for the array.

**Fig. 18:**
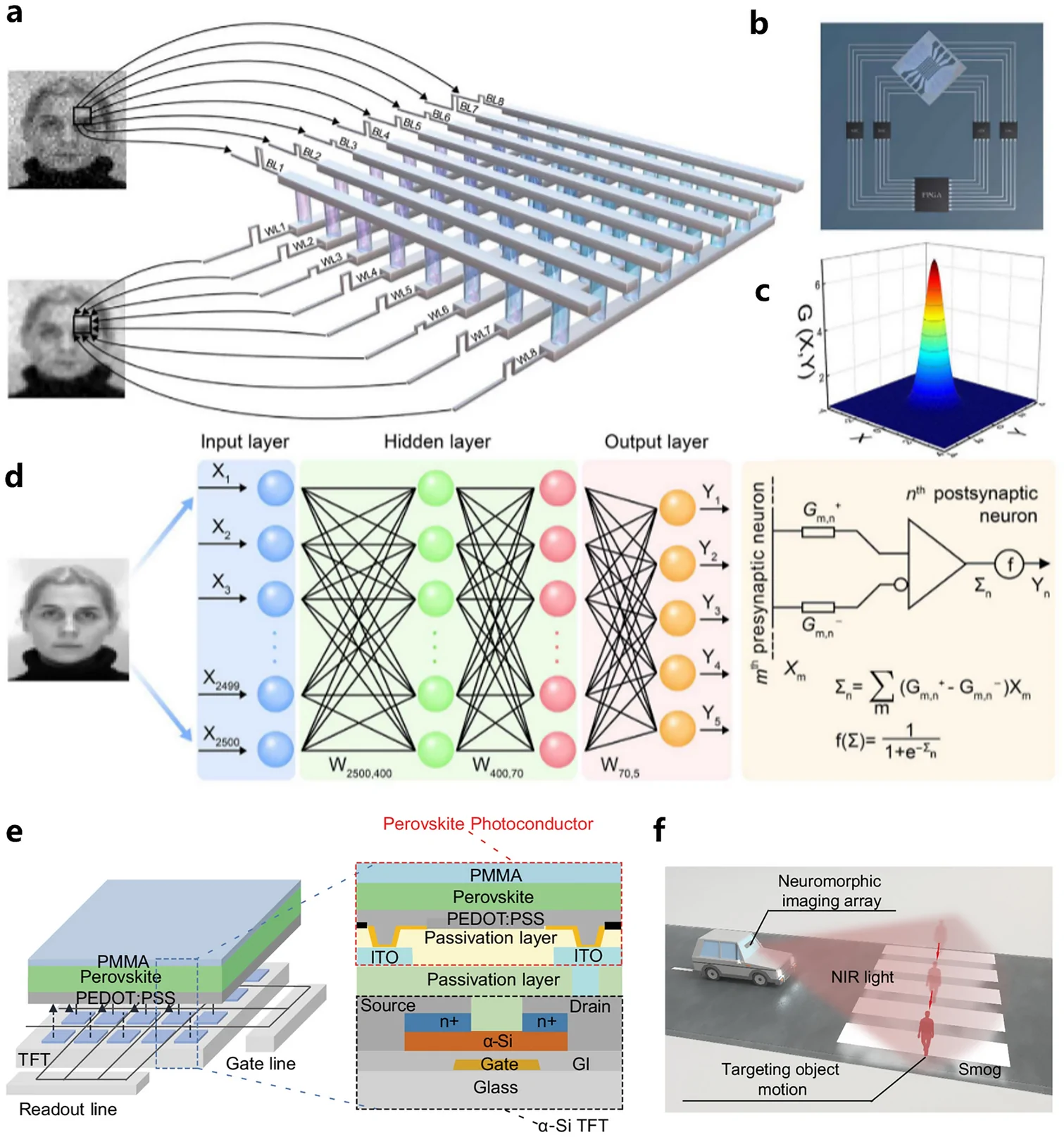
**a** Hardware image denoising system. **b** A schematic representation of the circuit block. **c** The Gaussian distribution applied during the denoising process. **d** The simulated neural network. Reproduced from Ref. [104] with permission from Elsevier, Copyright 2025. **e** Schematic structure of neuromorphic imaging array based on perovskite synapses. **f** Applications of neuromorphic imaging arrays in autonomous driving scenarios. Reproduced from Ref. [183] with permission from Springer Nature, Copyright 2025

Preliminary progress has also been made in hardware-level neuromorphic computing systems based on perovskite synapse arrays. As depicted in Fig. 18a, b, an 8 × 8 pixel memristive crossbar array was fabricated for executing the preliminary image denoising functionality [104]. The bit lines and word lines were interfaced with the STM32 processing unit via two digital-to-analog converters (DACs) and two analog-to-digital converters (ADCs), respectively. The input pulses, representing pixel values of the noisy image with varying amplitudes, are directly transmitted to 8-bit lines through STM32. The current outputs from the word lines are then collected and interpreted as the pixel values of the denoised image. Figure 18c presents the Gaussian distribution applied during the denoising process. Real-time visualization of the image denoising process was achievable through hardware. Subsequently, the pattern recognition task is carried out using the simulated ANN shown in Fig. 18d, and the denoising results are evaluated. The final recognition results indicate that the noise reduction process implemented by perovskite synapses has increased the recognition accuracy to approximately 94%. Furthermore, a neuromorphic vision imaging system based on perovskite optoelectronic synapse arrays has also been reported for in-sensor computation and spatiotemporal information fusion [183]. As shown in Fig. 18e, the perovskite-based 12 × 12 pixel neuromorphic imaging array is constructed on a TFT backplane. By integrating the TFT array with an image readout circuit chip, efficient image acquisition is realized, thereby realizing the heterogeneous integration of perovskite optoelectronic devices and silicon-based electronic circuits is enabled. Synaptic behaviors triggered by the NIR light source allow encoding of object motion through distinct photocurrent levels, thereby effectively integrating spatiotemporal motion information. The array can clearly visualize the contours of motion trajectories, holding great promise for applications in real-time object recognition in complex autonomous driving scenarios (Fig. 18f).

Despite significant progress in perovskite synapse arrays, a gap remains between these advances and practical neuromorphic vision computing systems. Extensive research efforts, from the materials level to the device level and to the system level, need to be carried out.

At the material level, the primary challenges lie in the uniform and clean deposition of perovskite materials, as well as the patterning of perovskite materials to facilitate device fabrication. Scalable, precisely optimized manufacturing methods are essential. The currently widely adopted approach offers advantages such as well-defined thickness and high quality. However, it is limited by substrate size and suffers from ink waste. Potential alternatives include vacuum evaporation deposition, blade coating and other related techniques [184]. In terms of the patterning of perovskite materials, potential solutions include template-assisted growth, substrate-assisted growth, capillary force-assisted growth, and direct patterning techniques [185]. As top-down approaches, the first three achieve patterning before the synthesis of perovskites, facilitating accurate control of the growth process for enhanced crystal quality [186]. In comparison, direct patterning techniques including photolithography and inkjet printing, allow patterning to be performed directly on perovskite thin films, exhibiting advantages of high resolution and precision [187]. It is worth highlighting that photolithography, the established industry standard and preferred technology, is typically incompatible with perovskite materials due to their chemical instability and sensitivity to wet chemical processing [188]. Thus, a crucial research direction lies in developing optimized photolithography techniques, such as the introduction of passivation layers and orthogonal lithographic processes.

At the device level, key aspects include structure design, downscaling, and performance scalability. Specifically, devices with shared bottom electrodes are essentially isolated, limiting their down scalability of the cell size. For 2 T devices, future research should focus not only on large-scale crossbar arrays but also on the impacts of sneak currents, wire resistance, and other related factors. To address this, engineering the units through an additional selector, diode and transistor has been proposed to attain enhanced control [189]. Self-rectifying resistive memory represent another solution without extra rectifying devices [190]. For 3 T devices, developing simple and universal integration methods for high density arrays is also imperative. Optimizing material deposition technologies combined with novel device structural designs, such as lateral-gate transistors, proves effective [191]. In terms of device performance, accurate characterization takes precedence, followed by performance enhancement. For the former, reliable protocols needs to be established [192]. For the latter, stability and endurance parameters of biomimetic devices need to be improved to meet reliability requirements. And performance metrics such as linearity and dynamic range must be maintained at high levels despite device downscaling [193].

At the system level, addressing these challenges requires cross-cutting optimizations across multiple aspects, including peripheral circuits, architectures, and algorithms. Fundamentally, developing energy-efficient and area-saving peripheral circuits that facilitate analog-to-digital conversion constitutes a significant challenge [189]. Possible approach involves on-chip peripheral circuits and fully analogue computing design to simplify and eliminate such conversion circuits [194, 195]. And 3D heterogeneous integration represents another potential direction, while further investigation from the circuit/architecture perspective still demands further research [196]. In addition to peripheral circuits, another critical bottleneck to overcome in architecture design is the efficient communication between synaptic-neuron cores. High-bandwidth interconnect structures, such as shared memory buses and networks-on-chip, can be employed to mitigate communication bottlenecks [197]. In addition, the co-design of learning and mapping algorithms with hardware is crucial. To mitigate limitations that are inherently challenging to solve with hardware advancements, it becomes imperative to develop strategic algorithmic at the appropriate abstractions levels for minimizing the overhead of learning energy and time [198].

In summary, due to the unique properties of biomimetic synapses, a fundamentally different approach is required to replace the traditional sequential development process. Moving forward, the ability to scale neuromorphic systems will require careful consideration of the interconnected multiple levels of materials, devices, and systems. Co-design and co-optimization are of great importance throughout development process of neuromorphic systems.

## Conclusions and Outlook

Given the impending explosion of sensory data in the era of big data and the Internet of Things, bio-inspired neuromorphic computing systems have attracted significant attention since their inception. It is widely recognized that biomimetic synaptic devices with sensing, memory, and computing capabilities are indispensable building blocks for such systems. Under the current landscape, the integration of perovskite materials not only enhances the performance of these devices but also expands their potential applications. The rich material library and excellent properties of perovskite materials have driven the emergence of a diverse range of synaptic devices. Generally, organic–inorganic perovskites characterized by low ion migration activation energy are often linked to 2 T devices based on ion migration mechanisms (including VCM and ECM). In contrast, inorganic perovskite quantum dots, which exhibit superior photosensitivity, are more favorable in devices that depend on the electron migration mechanism (including FG, HJ, DE, and FE). Moreover, the stimulation of perovskite synaptic devices using optical and/or electrical pulses has enabled the emulation of a wide range of synaptic behaviors, such as EPSC, PPF, and LTP. Such synaptic plasticity can be harnessed for diverse application scenarios, such as color recognition and image recognition, among others. Despite significant advancements in synaptic behavior simulation, neuromorphic applications based on perovskite synapses are still in their early stages. Research is currently limited regarding mechanistic studies, the hardware implementation of neuromorphic vision systems, and exploration of practical application scenarios, with several challenges yet to be addressed.

**Fig. 19:**
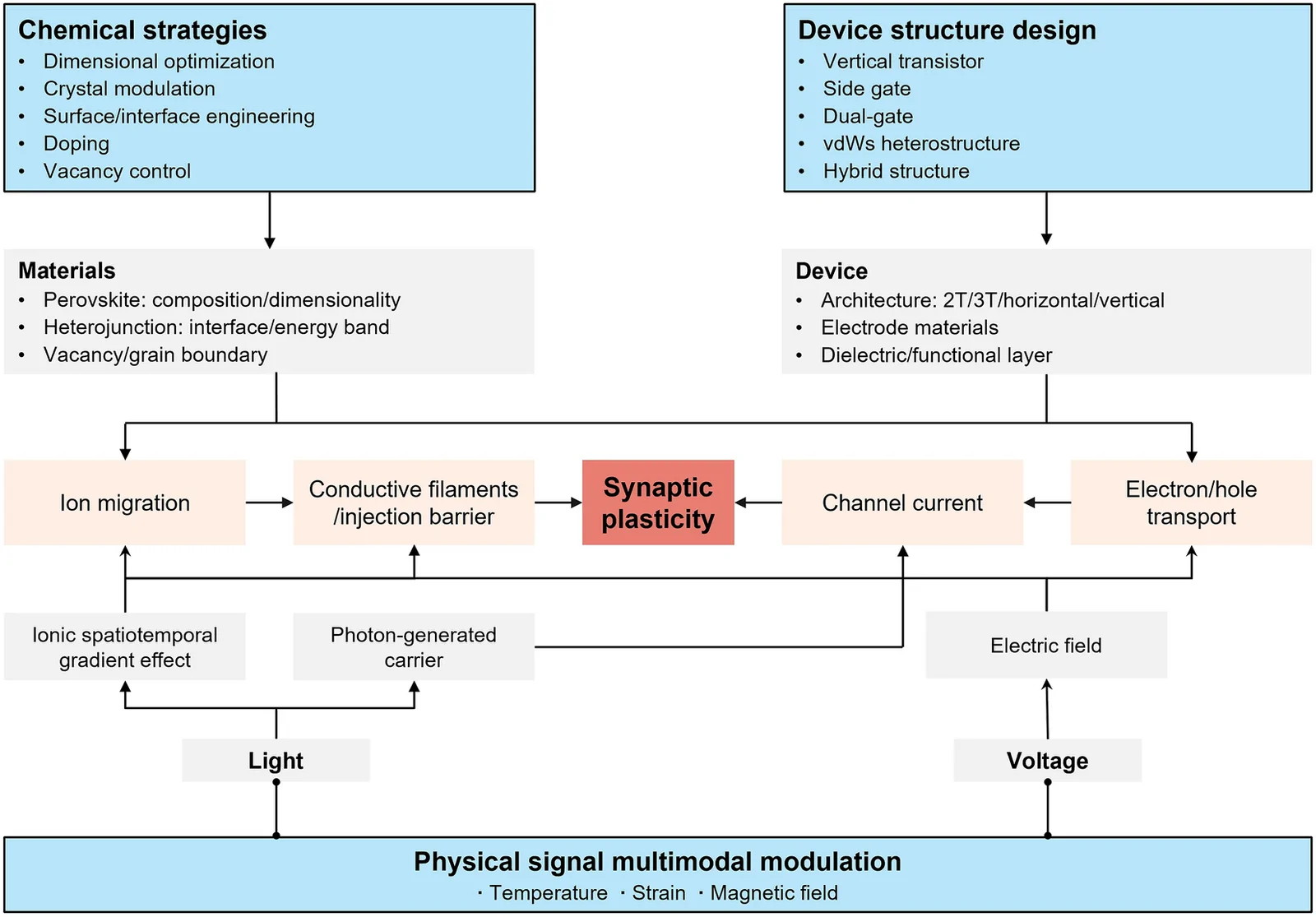
Multiple optimization opportunities for synaptic plasticity of perovskite devices

(1) For perovskite materials, as the fundamental basis for supporting device performance, not only their functionality needs to be evaluated, but their stability and potential toxicity necessitate meticulous evaluation and cautious handling. Most of the reported perovskite synaptic devices contain naturally toxic lead. With the growing prevalence and increasingly stringent environmental protection regulations, the application of Pb-based perovskites requires more comprehensive evaluation. Besides, the complex ion-electronic properties within perovskite materials also lead to insufficient understanding of the underlying mechanisms governing synaptic behavior. Further in-depth research should be conducted on the conditions triggering resistive switching to enable their reliable reproduction. Appropriate characterization techniques must be adopted to ensure the valid confirmation of the working mechanism. Recommended options include in situ and quasi-in situ methods, as well as simulated calculation approaches such as kinetic Monte Carlo and molecular dynamics.

(2) For perovskite synaptic devices, consistently improving the devices’ synaptic plasticity simulation performance is crucial for future advancement, with goals divided into two main aspects: enhanced computational capacity and advanced sensing ability. The potential methods are summarized as shown in Fig. 19, covering three key aspects: chemical strategies, device structure design, and physical signal modulation. To enhance computational performance, the implementation of both material innovation and device architecture optimization are recommend. The interplay between ionic and electronic properties needs to be carefully regulated. Factors that contribute to increased ion concentration, such as surface defects and grain boundaries, enhance the memristor behavior of the 2 T synaptic device but impede the operation of 3 T synaptic transistors. Chemical strategies for modifying the properties of materials, such as doping strategies, surface and interface engineering, and vacancy control, are recommended. It is also feasible to design side gate or dual-gate devices to facilitate structural innovation and enhance controllability. For advanced sensing ability, research should concentrate on the modulation of physical signals. Specifically, designing perovskite synaptic devices that can process high-dimensional information is beneficial. Also, there is a pressing need for multimodal collaborative modulation techniques to enable perovskite synaptic devices to achieve the integration and processing of multiple signals.

(3) For performance evaluation index, the performance assessment of perovskite synaptic devices faces the challenge of standardizing key metrics. Moreover, the differences among research groups in terms of material synthesis, device fabrication, and measurement protocols complicate the extraction of trends and comparative analysis. Despite these discrepancies, the reported device performance remains noteworthy. Advancing the field requires the concurrent reporting of key performance metrics, such as endurance, variation, and reliability, to comply with the best practices advocated in the literature. Furthermore, future work in this area should investigate the performance-to-area scaling trends to ascertain whether adequate performance can be retained upon downscaling.

(4) For the application prospects of perovskite synapses in neuromorphic vision computing, the current research is still in the initial stage. While perovskites are widely investigated for neuromorphic applications, multiple articles provide compact modeling based on their large-size devices, combined with neural network simulation platforms to demonstrate the application potential of their devices. This approach is prone to being inconsistent and misleading since constructing networks with thousands of synapses remains fraught with challenges. Future advanced applications not only require progress in regulating the synaptic plasticity but, more importantly, the realization of their hardware implementation and interconnection with other components. This calls for joint efforts in optimizing material compositions, advancing preparation methods, and developing patterning techniques. Once these key challenges are addressed, the simulation of large neural networks will grow more true-to-life, and the potential of neuromorphic applications will be more convincing.

In summary, the development of biomimetic perovskite synapses is inherently interdisciplinary, spanning fields such as materials science, biology, and computer science. Despite persistent challenges at the materials, device, and system levels, we remain optimistic that through concerted efforts by researchers across these disciplines, perovskite synapses can be leveraged to realize robust neuromorphic computing systems. We believe that perovskite synapses will play a crucial role in enabling high-fidelity neuromorphic vision computing systems to achieve unprecedented energy efficiency and throughput.
